# Integration of urban ecosystem-based adaptation in Nepal: A policy landscape analysis

**DOI:** 10.1371/journal.pone.0297786

**Published:** 2024-01-31

**Authors:** Tshering Ongmu Sherpa

**Affiliations:** Graduate School of Global Environmental Studies, Kyoto University, Kyoto, Japan; National Technical University of Athens: Ethniko Metsobio Polytechneio, GREECE

## Abstract

Ecosystem-based adaptation (EbA) is an ecologically sensitive, cost-effective, and locally adaptive climate adaptation strategy to strengthen the climate resilience of vulnerable communities. While many studies on EbA have been conducted in rural and mountainous regions or within the natural sciences realm, there is a lack of comprehensive research that assesses how urban EbA measures have been incorporated into existing policies and plans in Global South, including in Nepal. Ecosystem-based adaptation is in the early stages of its establishment as a fundamental component to address climate adaptation and sustainable development in urban environments. Accordingly, effective integration strategies, challenges, potential focal areas, and entry points have yet to be extensively studied. To address the literature gap, this paper analyses the types of EbA interventions and the extent of urban EbA integration within Nepal’s climate, urban, and sectoral policies and plans. Direct content analysis and a qualitative scoring system were used to evaluate the plan components and assess the level of EbA integration. The findings indicate that the policies and plans recognise the importance of conserving, enhancing, and managing ecosystems for climate change adaptation, and EbA measures are mainly included in action-oriented sections. However, the results also reveal inadequate EbA integration, particularly in the *information base*, *vision and objectives*, and *implementation* aspects. The *implementation* component notably lacks comprehensive provisions for budget allocation, responsible authorities, definite timelines, and clear roadmaps. The breakdown of EbA integration in the policies and plans suggests that climate and urban plans substantially integrate urban EbA measures, but discrepancies exist with climate and urban policies and sectoral policies and plans. These findings collectively emphasise a pressing need to enhance the recognition and integration of urban EbA measures within policy frameworks with a view towards strengthening climate resilience and mitigating climate-related hazards in urban environments.

## 1. Introduction

### 1.1. Concept of ecosystem-based adaptation

Ecosystem-based adaptation (EbA) is a climate change adaptation approach and sub-category of nature-based solutions which has gained traction in recent years for supporting sustainable urban development in the context of changing climatic conditions. The United Nations Convention on Biological Diversity defines EbA as ‘the use of biodiversity and ecosystem services as part of an overall adaptation strategy to help people to adapt to the adverse effects of climate change’ [1]. Ecosystem-based adaptation principally dovetails both ecosystem and human components and advocates for the acceleration of integrated climate actions rather than siloed approaches [2]. Practices of EbA range from preservation, restoration, and sustainable management of existing ecosystems to the creation of new ecological structures [3, 4]. Ecosystem-based adaptation has applications across a wide array of ecosystems, geographical regions, and sectors and amongst different stakeholders [5]. EbA often involves incorporating ecological principles into engineering practices and the resilience of the built infrastructures and canal systems depends on how well ecological considerations are integrated into its design and management as demonstrated in Kantartzis [6] and Mitoulis et al. [7]. The Sixth Assessment Report of the Intergovernmental Panel on Climate Change [8] recognises the importance of EbA in the urban environment and how its incorporation into conventional structural adaption responses may reduce adaptation costs and contribute to disaster risk reduction and management. Attention is gradually being directed to the remediation of urban areas to improve ecological and social health in conjunction with increasing climate resilience [9].

Urban EbA measures include strategies such as expanding green/blue infrastructures (e.g. green roofs and walls), urban green and open spaces, and urban agriculture and gardening. Green Infrastructure (GI), such as green roofs and facades, offers a two-fold advantage, providing both adaptation and mitigation benefits through carbon sequestration and reduced energy consumption. Structures like green roofs, are known for their advanced insulation that enhances building efficiency and results in emission reduction of greenhouse gases [10]. Additionally EbA measures like permeable pavements leverage on ecosystem services like soil permeability to combat climate-related hazards [11, 12]. These infrastructures transcend climate adaptation benefits [13] and offer numerous co-benefits, including biodiversity conservation, flood control, stormwater runoff reduction, urban heat island (UHI) reduction, carbon sequestration, food security, and recreation [14]. The bottleneck of extensive uptake of the EbA approach is the limited empirical evidence of its long-term adaptation benefits; still, a growing body of research has been validating it as a cost-effective alternative to traditional engineering-based approaches [15, 16].

### 1.2. Concept of ecosystem-based adaptation

There has been a relatively wide uptake and upscale of nature-based solutions and EbA applications in urban settings for climate change adaptation and mitigation in the Global North, where substantial evidence has been generated for EbA across the spectrum of green-grey approaches [11, 17, 18]. In contrast, scholarship linking urban EbA and climate adaptation [19, 20] has been scarce in the Global South [21]. Thematic areas encompassing biodiversity, agriculture, forestry, and disaster risk reduction have been prominently prioritised in past and ongoing EbA projects and literature [22–24]. Since EbA in urban settings is relatively new, there is only fragmented research on conceptualising and implementing urban EbA and its mainstreaming into policies [25–28]. To fully harness the latent potential of EbA, its integration into policymaking and planning is crucial to achieve sustainable and scalable interventions and ensure long-term effectiveness and resilience [29, 30]. Significant gaps in the literature include comprehensive research on the degree to which urban EbA approaches are integrated into current policies and plans and the most effective strategies of integration for identifying existing challenges, potential scopes, and strategic entry points [31].

### 1.3. Need for urban EbA integration in Nepal

Nepal is ranked fourth in the Global Climate Risk Index [32]. It is also amongst the least urbanised countries in the world. Yet, according to United Nations Department of Economic and Social Affairs [33], Nepal is projected to be one of the top ten fastest-urbanising nations from 2014 to 2050. The country’s urban population growth is sitting at 2.31% [34], and the federal restructuring of the governance system has caused an exponential increase in the proportion of urban residents to 66.17% of the total population [35]. Climate-related hazards are prevalent in both rural and urban areas, but the focus has been primarily on remote and rural places due to their underprivileged socio-economic conditions and geographically challenging topography. The lack of attention to urban areas may have detrimental effects in the near future, as the rate of urbanisation is rising rapidly in urban agglomerations, such as the Kathmandu Valley (comprised of the Kathmandu, Bhaktapur, and Lalitpur districts). The built-up area in the valley increased by 412% from 1986 to 2016 and is inhabited by 24% of Nepal’s total urban population [36].

The compound impacts of climate change and urbanisation, which include erratic rainfall, land use changes, and weak stormwater drainage systems, are causing a higher frequency of pluvial and fluvial flooding in Kathmandu Metropolitan City [37], which is inflicting harm on the public and resulting in property loss and disruptions to transportation. The city is also experiencing an increase in land surface temperature in core areas where concrete infrastructures are dense due to the high population concentration [38]. According to Ministry of Health and Population [39], the UHI effect is already inflicting adverse consequences on human health in the country’s urban areas. Failure to act poses a prompt and severe threat to urban dwellers, as the combination of climate change and rapid urbanisation will expose them to climate-related hazards, poverty, social inequalities, and other harmful effects.

In Nepal, EbA is a relatively novel concept. It can harmoniously complement engineering infrastructures and address the financial and specialised expertise demands often associated with conventional approaches. Thus, EbA represents a significant alternative method for Nepal, a country grappling with constant resource limitations, to galvanise its climate adaptation efforts [40]. Most EbA-related studies and projects have been conducted in rural and mountainous regions [40, 41]or in the natural sciences domain [42]. Currently, EbA is in a nascent stage of being established as a cornerstone of climate adaptation and sustainable development in urban spaces. In a study on climate change adaptation projects in Nepal from 2010 to 2020, Karki et al. [43] have reported that only 6 out of 76 climate change adaptation projects were dedicated to implementing EbA. An additional six projects were categorised as ‘EbA-related’ because they addressed capacity building, rural livelihood improvement, and knowledge management through integrating ecosystem and community-based approaches. The EbA-related interventions in Nepal have heavily relied on external support, which raises sustainability concerns regarding project outcomes after funding ends [43]. Previous successful pilot initiatives have not been adequately leveraged.

Few studies have conducted policy reviews to identify provisions for EbA mainstreaming in Nepal’s climate policies, plans, and programmes. Bhattarai et al. [44] have assessed the challenges of mainstreaming and upscaling EbA in developing countries based mostly on an empirical case study of Nepal’s Panchase Mountain Ecological Region. Their findings reveal that EbA interventions could effectively enhance socio-ecosystem resilience and reduce vulnerability; however, limited innovation, a lack of clear-cut policy arrangements, inadequate institutional mechanisms, and insufficient budget provisions can pose sustainability issues. Poudel et al. [45] have analysed climate change policies and the implementation of EbA-related activities in Nepal. Their results highlight the need to integrate EbA into regular planning processes, secure adequate funding, and establish robust institutional mechanisms for effective implementation and monitoring to overcome sustainability challenges.

These studies provide valuable insight into the state of the art of Nepal’s EbA initiatives, but they do not consider urban and sectoral policies and plans. Urban policies and strategies are believed to play a seminal role in maintaining and increasing ecosystem services to enhance liveability, improve sustainability, and build up the resilience of cities [46]. Sandholz’s [47] evaluation of the applicability of ecosystem-based approaches for disaster risk reduction in the Kathmandu Valley of Nepal has emphasised the importance of understanding human-nature interactions and the local value of natural assets in a setting characterised by rapid urbanisation, political instability, complex governance, and climate change impacts. It has further established a link between urban EbA strategies and climate adaptation but lacked a comprehensive review of relevant policies. To initiate and expedite EbA initiatives in urban settlements, national policies and policymaking processes should provide essential overarching guidance.

### 1.4. Research and purpose

In light of the identified gaps, this research first aimed to examine the types of EbA interventions and the level of EbA integration in Nepal’s climate, urban, and sectoral policies and plans. It sought to reduce the paucity of information by employing a qualitative scoring system adapted from previous studies [48] and performing an in-depth analysis of the plan components (*information base*, *vision and objectives*, *actions*, and *implementation*) to appraise the degree of integration. Second, the study aimed to identify strategic entry points for EbA in climate, urban, and sectoral policies, thus contributing to a more holistic and informed understanding of the level of EbA mainstreaming that is needed. The findings reveal practical implications for policymakers in regard to catalysing and enhancing the mainstreaming and implementation of EbA initiatives in urban environments, which can in turn enrich the efficacy of climate adaptation strategies and building climate resilience.

## 2. Methodology

### 2.1. Policy and planning instruments

To identify the types and degree of integration of EbA in addressing climate-related hazards and developing climate resilience, a comprehensive review was conducted of 4 policies and 10 plans, which included climate, urban, and sectoral policies and plans relevant to urban EbA. The majority of these policies and plans were formulated and/or revised after 2008, when the ‘ecosystem-based adaptation’ term was conceived, to ensure that there had been sufficient time to integrate EbA principles into the policies.

- Climate policies and plans: This category of policies is targeted to address and mitigate the effects of climate change at the national, provincial and local levels. It includes National Climate Change Policy (NCCP) 2019 [49], National Adaptation Programme of Action (NAPA) 2010 [50], Local Adaptation Plans for Action (LAPA) 2019 [51] and National Adaptation Plan (NAP) 2021–2050 [52].
- Urban policies and plans: This includes policy documents relevant to urban development and planning that serve as guiding frameworks to shape the urban landscape and improve the quality of life of the urban residents at the national level and also specific to Kathmandu Valley. It comprises of National Urban Policy (NUP) 2007 [53], National Urban Development Strategy (NUDS) 2016 [54], Vision 2035 and Beyond: 20 years Strategic Development Master Plan (SDMP) 2015–2035 for Kathmandu Valley [55] and Risk Sensitive Land Use Plan of Kathmandu Valley (RSLUP) 2016 [56].
- Sectoral policies with urban EbA component: This category encompasses sector-focused policies that have a component related to urban EbA or ecosystem services. It includes National Environment Policy (NEP) 2019 [57], Disaster Risk Reduction National Strategic Action Plan (DRRNSAP) 2018–2030 [58], National Biodiversity Strategy and Action Plan (NBSAP) 2014–2020 [59], National Land Use Policy (NLUP) 2015 [60], Forest Sector Strategy (FSS) 2016–2025 [61], Water Supply, Sanitation and Hygiene Sector Development Plan (WSSHSDP) 2016–2030 [62].

### 2.2. Types of EbA measures

As a result of climate change, urban areas are becoming increasingly vulnerable to climate-related hazards, such as flooding and the UHI effect [3]. Informed by the works of Gómez-Baggethun and Barton [63] and Geneletti and Zardo [64], this study investigated the EbA measures present in the analysed policies and plans, which can be broadly classified into two categories: (i) storm management, flood risk management, or water security; and (ii) temperature regulation. The study also considered EbA measures addressing the two categories of ecosystem services in general terms, without an urban focus, since the integration of urban EbA was not prevalent in all of the climate, urban, and sectoral policies and plans of Nepal that were selected for appraisal.

The existing scholarship does not provide a standard list of urban EbA measures. However, past studies have adopted and revised the classification proposed in the European Environment Agency (EEA) [65]. The EEA list was formulated for European cities, where the foundation of EbA and its umbrella concept, nature-based solutions, is far more advanced, and the level of EbA integration in policymaking is considerably greater than in Nepal. The list of EbA measures used for the analysis in this paper was collated from the plans and policies. Table 1 presents an overview of the EbA measures derived from the analysis of the plans and policies, which aligns with the identified categories. The EbA measures were further characterised as general EbA measures or urban EbA measures. The general classification may include urban components, but the urban category consists exclusively of EbA measures from an urban context. With reference to Zölch et al. [18], the urban EbA measures include components of the urban ecosystem, such as urban biodiversity and green/blue infrastructures (parks, urban forests, and rivers, streams, and lakes, as originally classified by Bolund and Hunhammar [66]) together with the greening of buildings and vegetation along the streets and in gardens (elements added to green infrastructures in the work of Benedict et al. [67]).

**Table 1:** EbA measures identified from the plans and policies.

EbA measures	Type
Develop green belts and open spaces alongside rivers, roads and canals	General
Protect, develop, promote and manage forest	General
Protect water bodies-ponds, wells, rivers and canals	General
Integrated watershed management to protect and regenerate/rehabilitate degraded water bodies, watersheds, lakes, wetlands and ponds	General
Redevelopment of river bank into open spaces for flood-prone area	General
Managing flood plains and stabilizing river banks using forest	General
Promote rainwater harvesting and increase groundwater recharge through conservation ponds (reservoirs) and contour ditches and rehabilitation of traditional ponds for protection of natural infiltration zones	General
Conservation of the ecosystem and biodiversity services	General
Conserve, develop and promote greenery (e.g. green parks and green open spaces)	Urban
Protect urban biodiversity through landscape conservation and climate resilience approaches	Urban
Promote urban forest and sustainable management of urban forest	Urban

### 2.3. Evaluation of EbA measures in the policies and plans

In line with the existing literature on appraising policies for the uptake of EbA and the ecosystem services concept, this study systematically analysed the policies and plans to identify explicit or implicit use of EbA measures across four plan components: *information base*, *vision and objectives*, *actions*, and *implementation*. The classification was adapted from Heidrich et al. [68], Geneletti and Zardo [64], and Cortinovis and Geneletti [48] as a modified version of the classification proposed by Baker et al. [69]. This modified version excludes the fifth plan component, options and priorities, since there was inadequate information on this component in the selected plans and policies. For this paper, *information base* included any statements indicating cognisance of general or urban EbA measures concerning storm management, flood risk management, or water security and temperature regulation. *vision and objectives* referred to statements specifying goals associated with the application of such EbA measures (qualitative or quantitative). *Actions* encompassed any specific actions undertaken to employ general or urban EbA measures, including strategies and projects. Finally, *implementation* included any provisions related to general or urban EbA measures intended to guide the enforcement of actions (e.g. budget details, responsible authorities, project duration).

**Fig 1:**
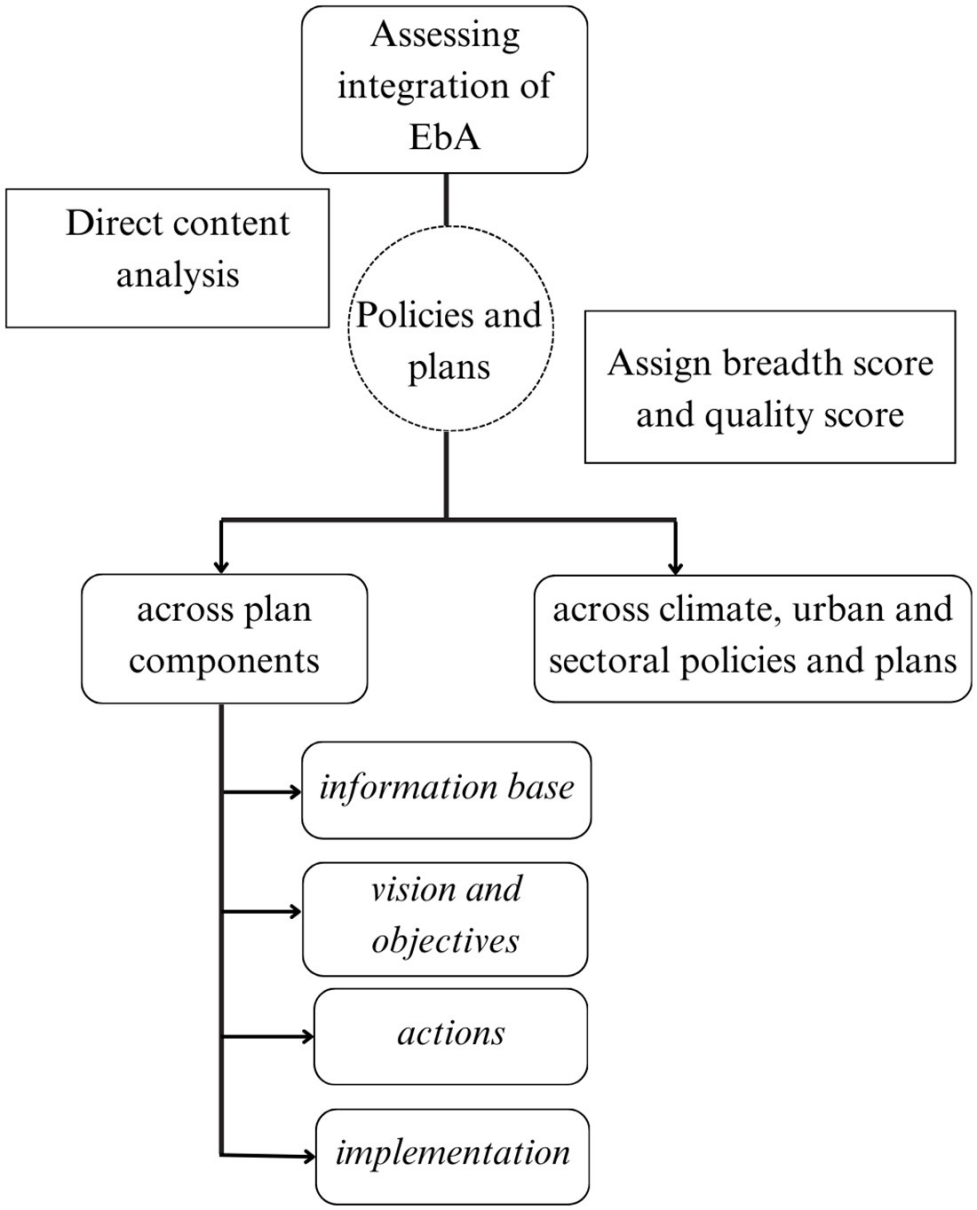
Framework to assess the integration of EbA.

Direct content analysis was conducted to review the documents and assess the inclusion of the EbA measures listed in Table 1 in two domains: (i) across each of the four plan components and (ii) across different climate, urban, and sectoral plans and policies. The diagrammatic illustration of the framework adopted to assess the EbA integration is provided in Fig 1. Previous studies have used this type of content analysis to examine the integration of EbA into urban planning documents and landscape plans [28, 48] as well as urban climate adaptation plans [64]. It has also been used to assess the integration of ecosystem services into strategic environmental assessments across, for instance, spatial plans and the transfer of development rights [70, 71].

Direct content analysis employs existing theories or prior research in a deductive manner. The first step is to identify key concepts and variables [72], which serve as the basis to define coding categories and operational definitions drawn from the existing literature. Direct content analysis is preferred over summative (keyword-based) content analysis here since EbA is not yet a standardised term that has been extensively incorporated into policy documents [73]. The analysed policies and plans use a wide array of terminology to denote EbA measures. All four plan components were considered when evaluating the plans, which provide detailed roadmaps for achieving specific goals by outlining a comprehensive programme of actions. However, the evaluation of the policies only took three plan components into account, with *implementation* being excluded. This choice was made because the policies primarily offer guiding principles or guidelines for the actions of the concerned authorities.

### 2.4 Assessing the breadth and quality of inclusion across plan components and policies and plans

The breadth score indicator was measured to capture the distribution and extent of EbA measures within the analysed plans and policies. This indicator was initially introduced by Tang et al. [74] and subsequently applied by Kumar and Geneletti [75] and Cortinovis and Geneletti [48] in their studies on the integration of ecosystem services into urban plans. The indicator evaluates the proportion of plans and policies that incorporate content pertaining to EbA. The present study is solely concerned with the breadth score. The depth score was omitted because its calculation necessitates the presence of non-zero components in the plans. This criterion was not fulfilled by the data in this study, as some policies and plans made no mention of EbA in their components and therefore had a score of 0. This study adopted a qualitative scoring protocol to measure the quality of Nepal’s climate, urban, and sectoral plans and policies concerning the inclusion of EbA measures. This scoring system provided a framework to evaluate the degree of EbA integration as well as a standardised approach to assess and compare the level of inclusion across multiple plans and policies. The scoring system was developed with reference to Geneletti and Zardo [64] and Schneider et al. [28], whose work mainly assesses the inclusion of urban EbA measures in the climate adaptation plans and landscape plans of European cities. These two studies based their scoring criteria on the extent of information related to urban EbA. The current study differs in that the highest score was assigned to policies and plans with urban EbA inclusion. The assessment involved determining the presence or absence of EbA measures and assigning a score ranging from 0 to 3 to indicate the degree of integration across various plan components. The scoring protocol for this study used a four-point scale, whereas previous studies utilised a five-point scale [69, 71]. This decision was justified by the relatively nascent nature of EbA as a concept in Nepal and its consequently limited incorporation into policies and plans. A four-point scale was deemed more appropriate to evaluate the degree of integration since policies and plans may not address the topic comprehensively enough to warrant a more intricate scoring system. Tables 2 and 3 display the scoring criteria for measuring the level of EbA integration to comprehensively evaluate the treatment of EbA measures in each policy or plan. The research scores underwent a cross-check process carried out by two additional researchers, who were briefed on the research objectives and scoring guidelines. These two researchers and the author each individually rated the plans and policies using the scoring protocol to assess the inter-rater reliability, ensure consistency, and identify any potential scoring bias introduced by the author. The obtained intraclass correlation coefficient exceeded 0.7, which met the criteria for acceptable reliability according to Koo and Li [76]. This result indicates that the scoring process was robust, and any potential bias was effectively minimised.

**Table 2:** Scoring protocol for integration of EbA measures in *information base* and *vision and objectives* plan components (Adapted from Geneletti & Zardo [64] and Schneider et al., [28]).

Score	Information base	Vision and Objectives
0	Absence of information regarding EbA measures	Absence of clear objectives or goals related to EbA measures
1	Introduces EbA measures but not in the context of mitigating climate-related hazards	Mentions utilizing ecosystem services in the objectives without specifying any EbA measures
2	Includes applying EbA measures to address climate-related hazards	Mentions utilizing specific EbA measures in the objectives but not in urban context
3	Includes applying urban EbA measures to address climate-related hazards	Mentions utilizing urban EbA measures in the objectives

**Table 3:** Scoring protocol for integration of EbA measures in *actions* and *implementation* plan components (Adapted from Geneletti & Zardo [64] and Schneider et al., [28]).

Score	Actions	Implementation
0	Absence of action-oriented activities or information on application of EbA measures	Absence of implementation provisions related to EbA measures
1	Consists of information implicitly referring to EbA in the actions	Comprises of EbA-related implementation provisions without providing further details
2	Consists of specific EbA measures in the actions but not in urban context	Comprises of EbA-related implementation provisions and provides details, including budget, responsible bodies, and project duration
3	Consists of urban EbA measures in the actions and provides information on their application	Comprises of EbA-related implementation provisions and provides details, including budget, responsible bodies, project duration, along with defining the approach for inter-sectoral and inter-ministerial collaboration

## 3. Results

### 3.1. Breadth of EbA inclusion in plans and policies

**Fig 2:**
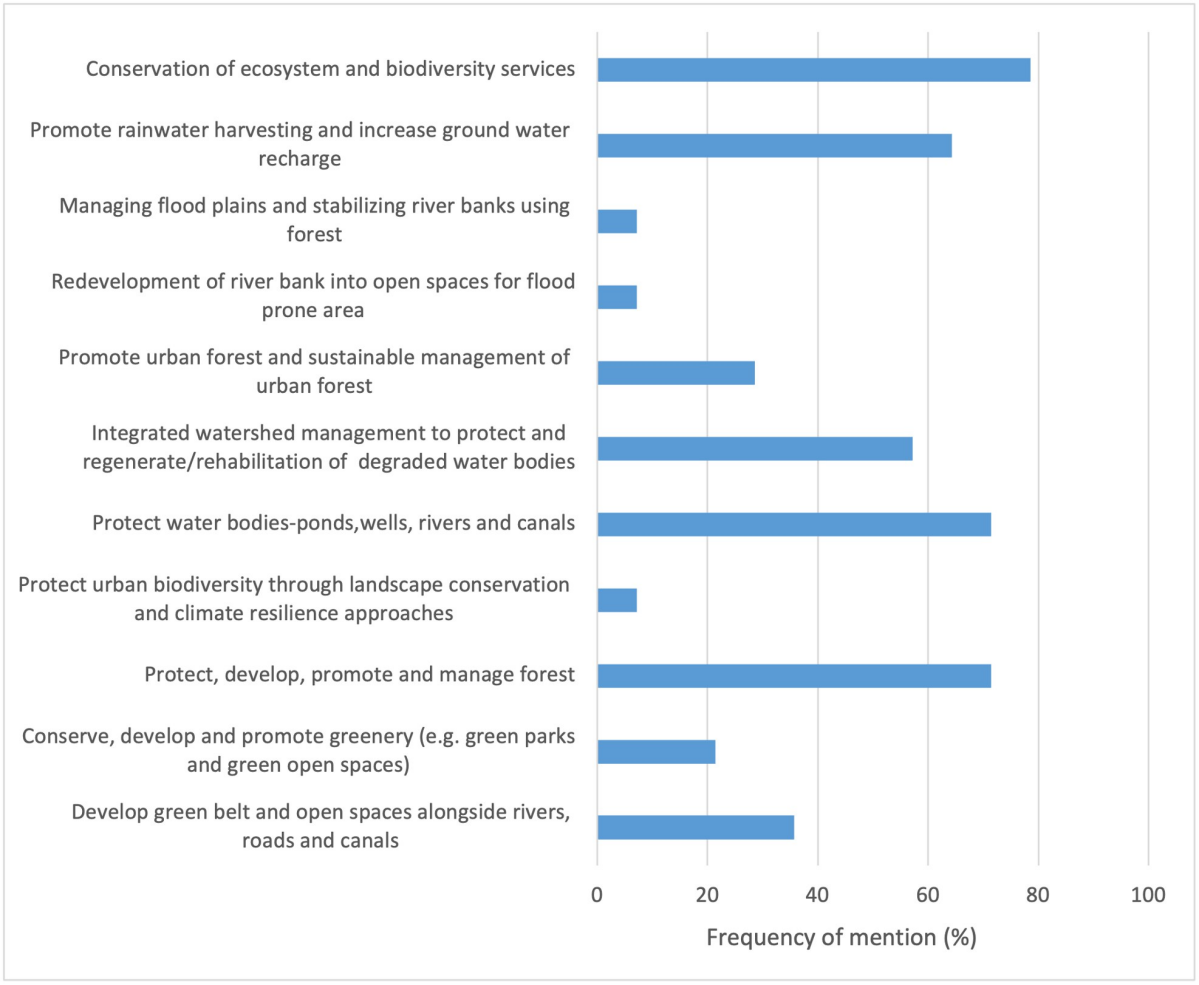
The variation in breadth score indicator value across EbA measures in any one of the plan components.

The conservation of ecosystems, including forests, water resources, and ecosystem services, had high coverage, with more than 70% of the plans and policies addressing it (11 out of 14; see Fig 2). Interventions related to rainwater harvesting and integrated watershed management had the second-highest coverage, with a presence in over 55% of the plans and policies (9 out of 14). Meanwhile, urban components, such as urban forests, urban biodiversity, green open spaces, and green parks, had limited coverage of less than 30% in the plans and policies. Urban heat island measures were largely missing from all plans and policies.

### 3.2. Frequency distribution of EbA measures

**Fig 3:**
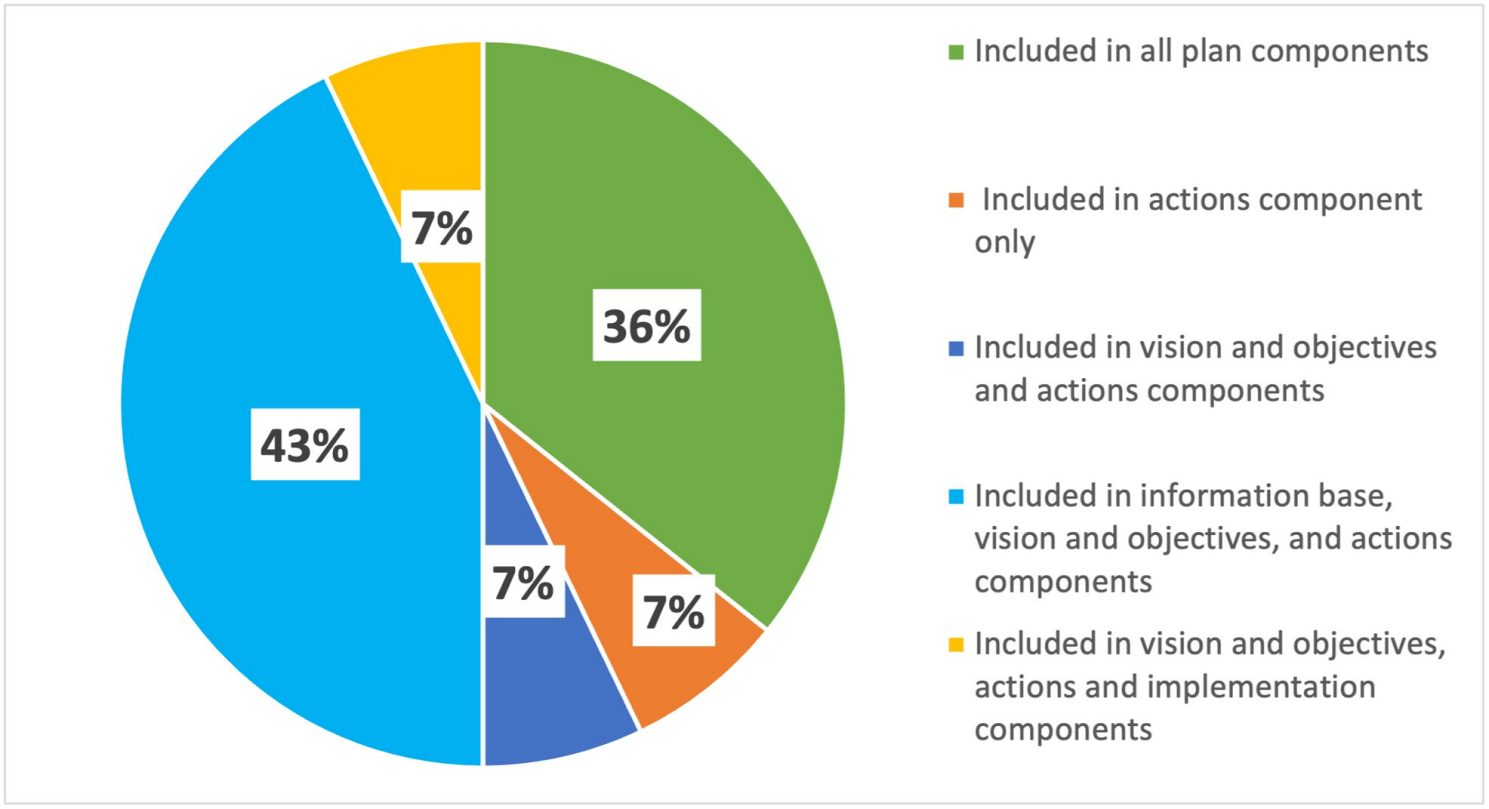
Distribution of information regarding the identified EbA measures among the four plan components.

Only 36% of the examined plans and policies incorporate EbA-related information within at least one plan component (Fig 3). Amongst this subset, 43% of the plans and policies contain EbA-related information within the *information base*, *vision and objectives*, and *actions* components but lack corresponding linkages in the *implementation* component. Approximately 7% of the plans and policies address EbA in the *actions* component only. The same percentage address it in both the *vision and objectives* and *actions* components or explicitly integrate it in the *vision and objectives*, *actions*, and *implementation* components.

**Fig 4:**
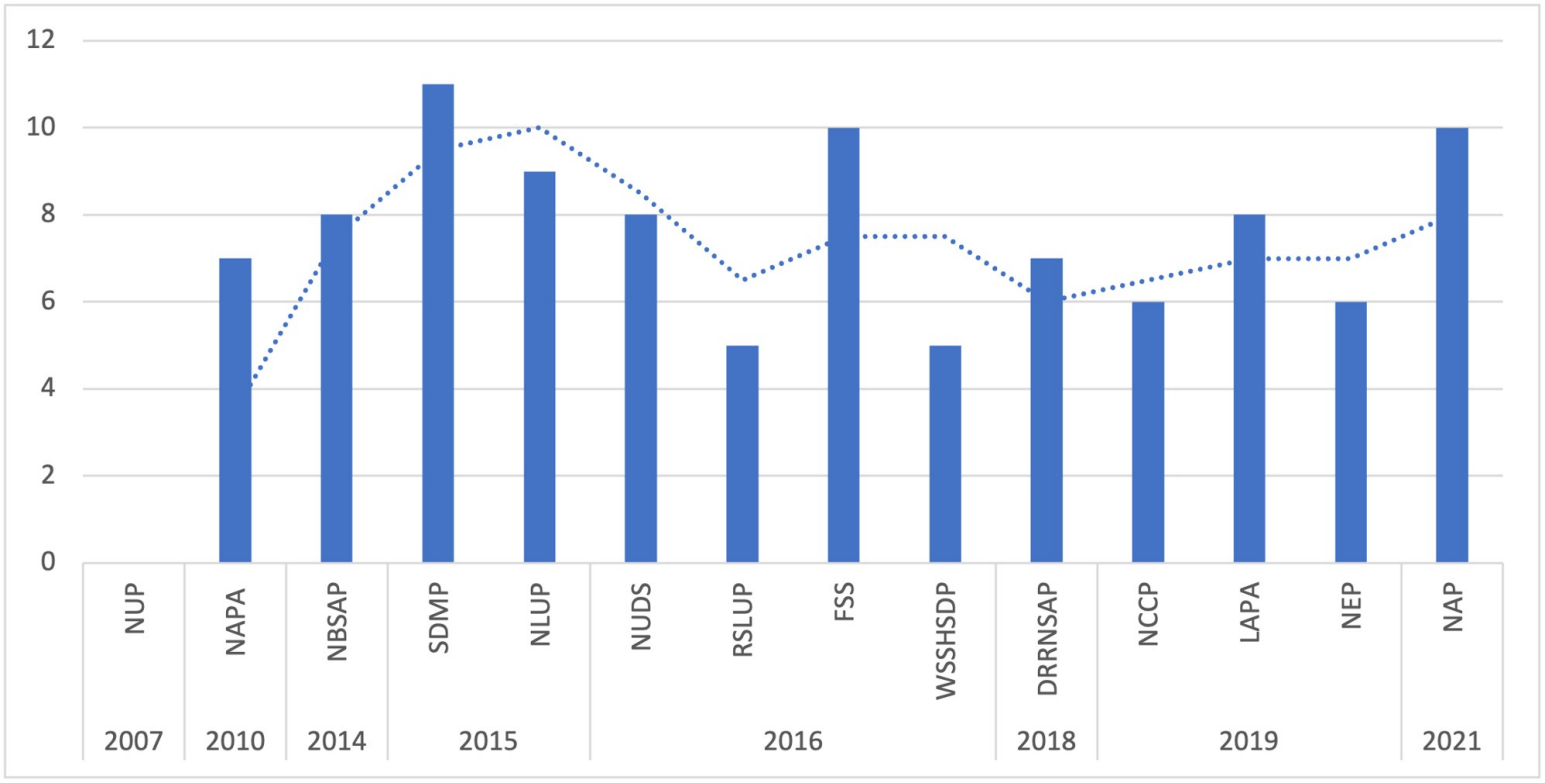
Frequency of information related to EbA measures across plans and policies.

The analysis of the climate change plan documents, including NAPA (2010), LAPA (2019), and NAP (2021), revealed a gradual increase in the number of mentions of EbA measures over time (Fig 4). As for the urban policies and plans, the SDMP for the Kathmandu Valley (2015) has the highest number of mentions related to EbA measures, while NUP (2007) has no explicit references to EbA measures. Concerning the sectoral policies and plans, FSS (2016) has the highest number of mentions amongst sectoral policies and plans, while WSSHSDP (2016) has the fewest mentions.

### 3.3. Breakdown of plan components across types of EbA measures

**Fig 5:**
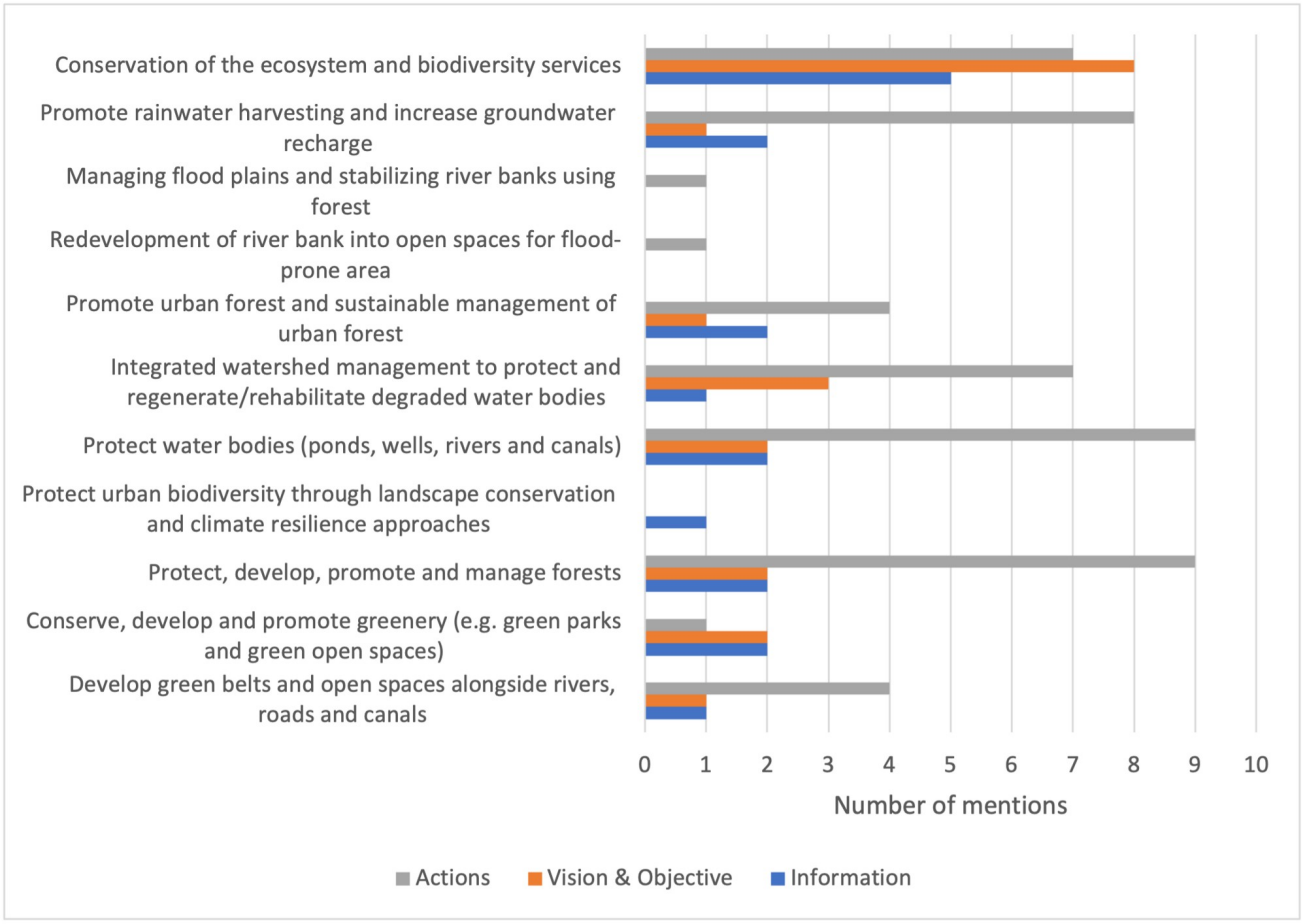
Distribution of mentions of the identified EbA measures across plan components.

Out of the 11 listed EbA measures, 10 measures address the *actions* component, 8 measures address the *information base* component as it specifically relates to EbA measures, and 9 measures address the *vision and objectives* component (Fig 5). Urban-specific measures, such as those regarding urban forests and green open spaces, have fewer mentions overall. Still, they are represented across all three components, with the exception of urban biodiversity, which is solely addressed with the *information base* component.

### 3.4. Quality scores across plan components and plans and policies

**Fig 6:**
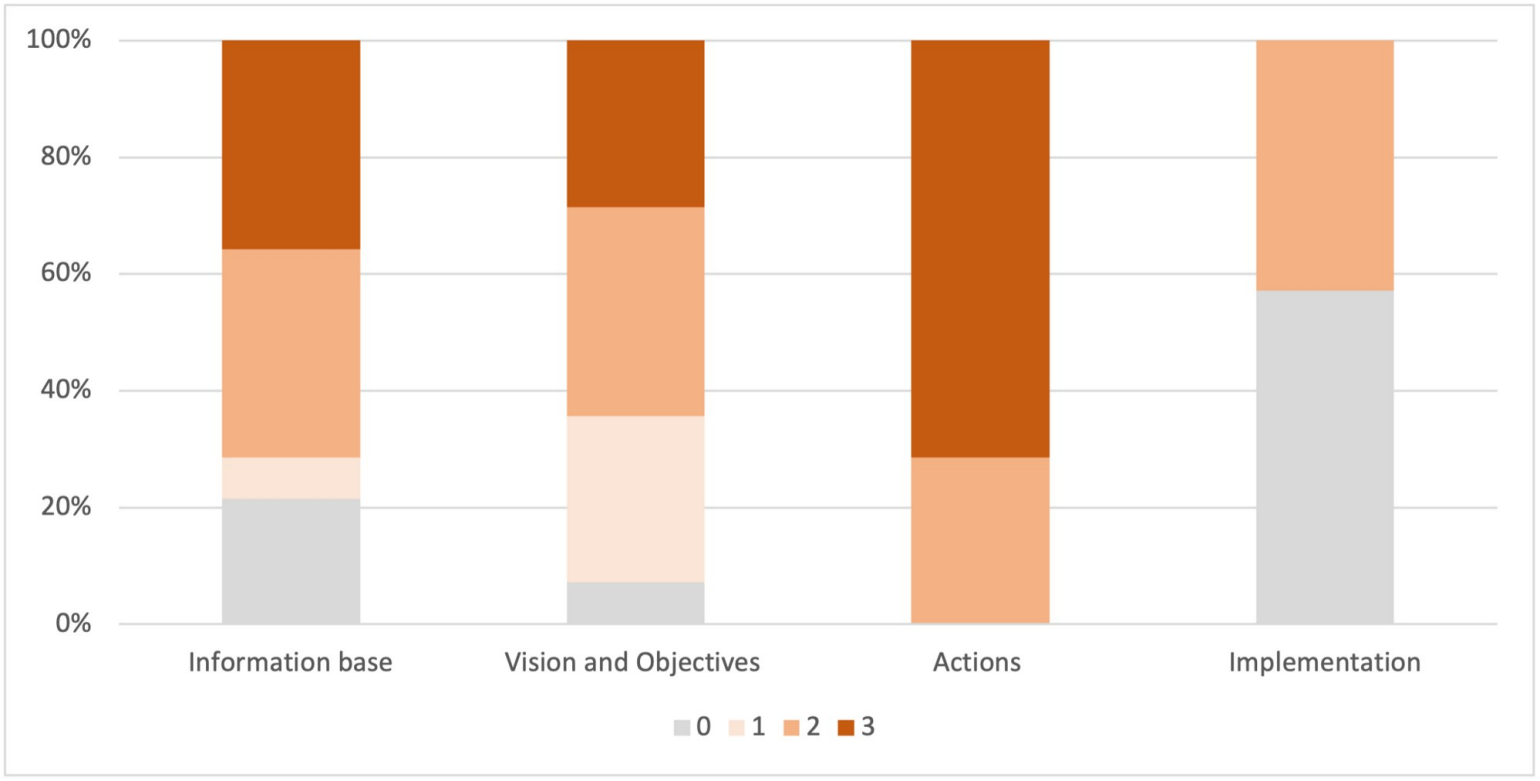
Distribution of quality scores in plan components for identified EbA measures.

**Fig 7:**
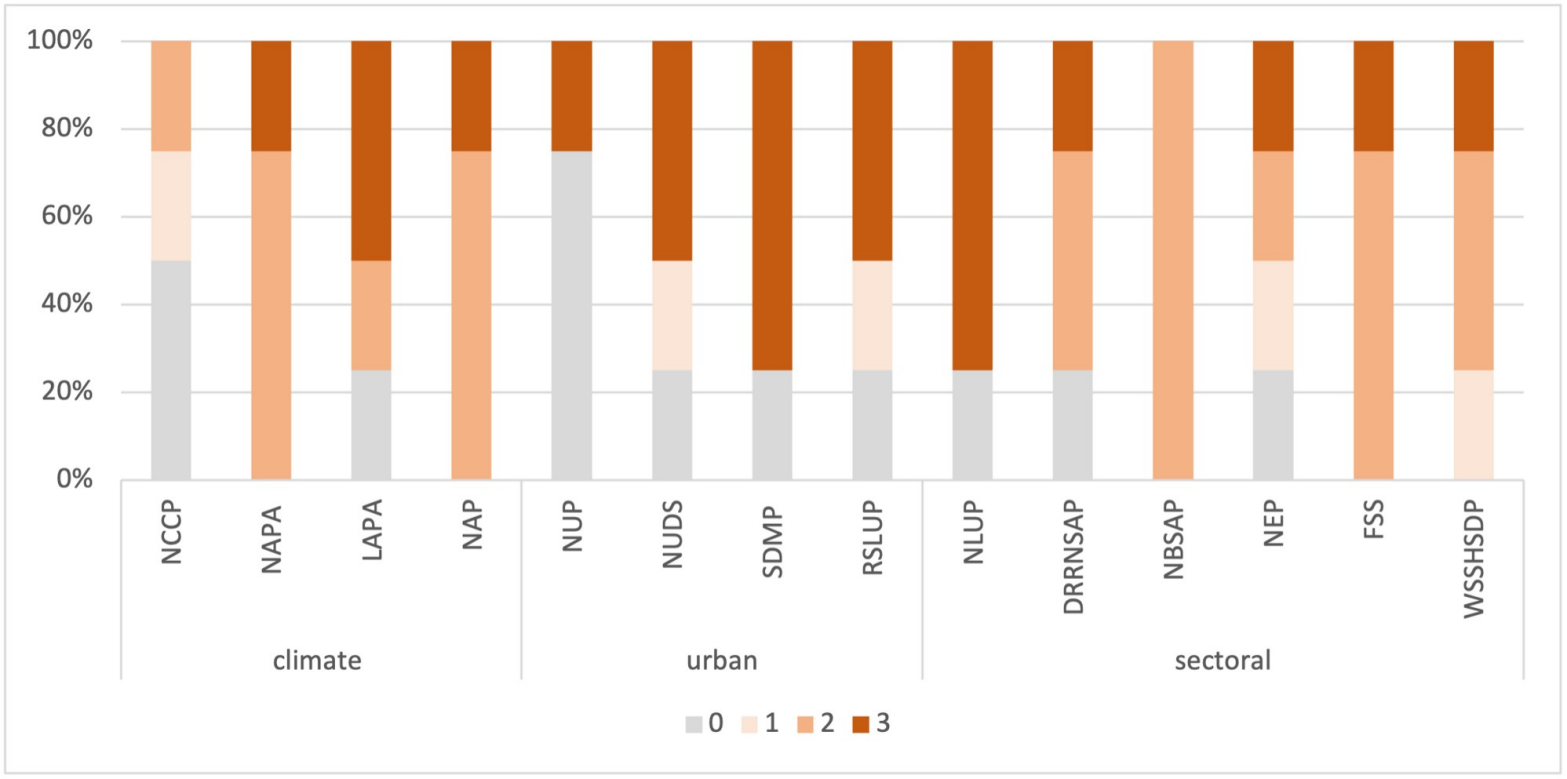
Distribution of quality scores in plans and policies for identified EbA measures.

The most common quality scores are 2 and 3 for the *information base* component (36% coverage each), 2 for the *vision and objectives* component (36% coverage), 3 for the *actions* component (71% coverage), and 0 for the *implementation* component (57% coverage; (Fig 6). The urban EbA concept is recognised across all plan components at rates ranging from 29% (*vision and objectives*) to 71% (*actions*). As for the distribution of quality scores across plans and policies, the most common quality scores are 2 and 3, which have coverage ranging from 25% to 100% and 25% to 75%, respectively (Fig 7). Additionally, 12 of the 14 analysed plans received a score of 3, which signifies an explicit reference to urban EbA in the plan or policy. Amongst these, NUDS, SDMP, RSLUP, and NLUP have 50% or higher inclusion. However, in the climate change policy and plans as well as the sectoral plans, particularly DRRNSAP, NEP, FSS, NBSAP, and WSSHSDP, there is either very little or no mention of urban EbA.

**Fig 8:**
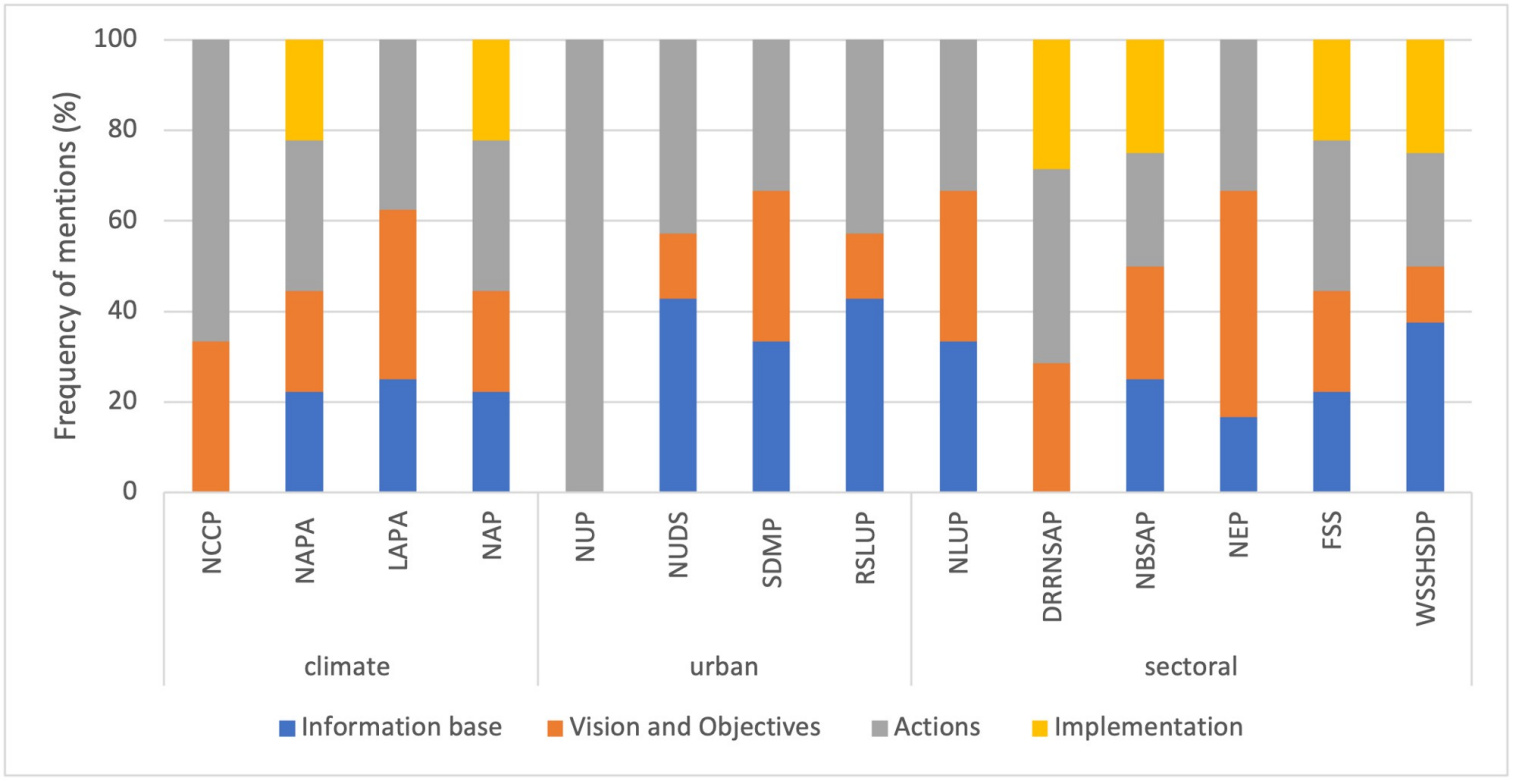
Distribution of information relevant to the plan components in the sample of plans and policies.

The distribution of the plan components is relatively uniform in four of the climate and sectoral planning documents (NAPA, NAP, NBSAP, FSS; (Fig 8). NUP includes EbA-related content in the *actions* component, while urban plans emphasise the *information base* component, accounting for a weightage range of 33% to 43%, but lack any integration of EbA-related information in the *implementation* component. In contrast, the climate and sectoral plans exhibit some degree of integration of EbA in the *implementation* component.

## 4. Discussion

### 4.1 Assessing the breadth of inclusion of EbA measures

Conserving ecosystems and biodiversity and ensuring a steady flow of ecosystem services are well addressed in most plans and policies. This finding indicates that the policymakers understood the intrinsic and utilitarian value of ecosystems and biodiversity. In addition, the concept of ecosystem services is incorporated to a substantial extent at the policy and planning level in dealing with climate change impacts, which is in line with the findings of Bouwma et al. [77]. However, the same cannot be said for the EbA concept in urban scenarios, as only three EbA measures are specific to urban areas. These findings underline the lack of attention and references to urban EbA measures in most of the plans and policies. This finding reaffirms that urban EbA is still in an early phase of development in Nepal, as observed by Huq et al. [31], who has reported that the role of ecosystem services in sectors such as urban planning, biodiversity management, and disaster risk reduction was not adequately addressed in Bangladesh, with a strong emphasis on prioritising and investing in grey infrastructures for climate adaptation. A clearer path and legislative framework are required to systematically embed the EbA concept into urban planning [78, 79], which underscores the need for a call to action to facilitate the uptake of EbA in urban policies and planning tools spanning various sectors.

While conservation-based measures are more commonly addressed, there are also mentions of other types of measures associated with the formation of new ecosystems (e.g. developing forested areas and green open spaces), rehabilitation (e.g. regenerating water bodies and watersheds), and the enhancement of existing ecosystems or ecosystem components (e.g. sustainable management and maintenance of forests). Zölch et al. [18] have also identified the four categories of EbA measures in the adaptation strategies of German municipalities. However, the presence of different types of EbA measures does not necessarily imply their in-depth and extensive integration, as evidenced by the quality score discussed in Sections 4.2 and 4.3.

The EbA measures linked to temperature regulation or reducing the UHI effect are limited. Although the measures listed in Table 1 have synergies and overlap in their benefits for combatting climate-related hazards, including flooding and urban heat stress, the absence of their explicit acknowledgement suggests that little significance is ascribed to this issue. A surge in land surface temperature has been reported in the capital city of Nepal, Kathmandu, mainly in densely populated urban areas where concrete infrastructures cover the majority of open and green spaces [38]. Adverse consequences for human health have already resulted from the UHI effect in the country’s urban areas [39]. The insufficient recognition of the thermal comfort afforded by green infrastructures in the *information base*, *vision and objectives*, and *actions* components is disconcerting. This gap should be filled by introducing and incorporating the aforementioned measures, including green walls and vegetated facades, especially in urban policies and plans. Such green and blue infrastructures cool the local microclimate by improving the thermal performance of buildings and enhancing cold air ventilation and cold air formation zones [80, 81]. The findings from Ribeiro [82] emphasized the need to tailor strategies according to the distinct urbanization patterns of cities and incorporate local knowledge, a crucial step in ensuring their long-term sustainability. Hence, to ensure the adaptive measures are sensitive and responsive to the impacts on the ground, the integration of climate science and the inclusion of multi-stakeholder engagement during the design and implementation of EbA measures are prerequisites [83].

### 4.2 Evaluating the integration of EbA measures across plan components

The disaggregated data on the inclusion of the EbA measures across the four plan components illustrate that the measures were not consistently incorporated into the plans and policies. This gap was principally due to a lack of exhaustive information about strategic plan components, including the *information base*, *vision and objectives*, and *implementation* components. Of these three, the *implementation* component was particularly neglected, with limited implementation provisions and details on operationalising EbA measures, including budget allocation, responsible bodies, and project duration. This finding aligns with those of Cortinovis and Geneletti [48] and Bhattarai et al. [44]. A robust implementation plan can serve as a roadmap and pragmatic basis for enforcing the set objectives and actions. Successful implementation requires a clear designation of roles, responsibilities, resource allocation and mobilisation, and monitoring and evaluation [84], which is largely missing in Nepal’s scenario.

Nepal underwent a historic transition when restructuring its governance system from a unitary state to a federal system in 2015 [85]. However, because of the complexity surrounding shared jurisdictions, there is an unclear division of roles and responsibilities both within and between governmental bodies at the federal, provincial, and local levels. This situation has led to low institutional ownership, duplication of efforts, and squandering of resources. For example, the Ministry of Forest and Environment is designated as the authority responsible for overseeing the functional coordination of NCCP and NAPA, but the local government is accountable to the Ministry of Federal Affairs and Local Development (MoFALD). There has been contention over the local government often presenting itself as an extension of MoFALD rather than an independent local governing body. MoFALD administers local governance and may not consider climate change within its purview, which potentially dissuades other ministries from delegating local government to carry out initiatives related to climate change [86].

Additionally, there is a discrepancy between the authorities granted and the institutional capabilities and capacities of provincial and local governments to implement the policies and plans [87]. The 2015 Constitution of Nepal establishes a framework for intergovernmental relations that allows the federal government to guide or assist local governments directly or through provincial governments. Despite these mechanisms, local governments often rely heavily on the federal government due to disparities in resource distribution, imbalances in functional and budgetary authority at the provincial and local levels, and high fiscal dependency. Various institutional mechanisms, such as inter-provincial councils and fiscal management acts, aim to mitigate these challenges; still, discrepancies persist and hinder effective collaboration between local and provincial governments. Therefore, the plans should elucidate implementation procedures to provide operational clarity for relevant ministries and different tiers of government. While this study reveals a biased inclination towards the *actions* component, directing more attention to the *implementation* component is equally crucial to translate ambitious action targets into tangible outputs and outcomes.

As mentioned, the *actions* component has more mentions of EbA and a larger share of plans and policies encompassing information about urban EbA applications compared to the other three plan components. This finding implies a strong focus on operationalising the targets of the policies and plans and a heavy emphasis on actionable strategies. This trend corresponds with the finding of Geneletti et al. [88] that the *actions* component reflected EbA measures to a greater degree than the other components. However, the breakdown for each of the EbA measures reveals a disparity in the inclusion within the *actions* component, as some measures are mentioned considerably more often than others. In line with the observations for the value of the breadth score indicator, the actions are more concentrated on protecting water bodies and forests, promoting sustainable forest management and integrated watershed management, and using rainwater harvesting technology to enable onsite filtration and retention of rainwater. The urban EbA measures receive little attention, which is echoed in a study by Sandholz et al. [19] where EbA-centric approaches to adapting to climate change risk were more prevalent in rural areas than in urban ones. The lack of attention to the urban ecosystem and its biodiversity is alarming since it could hinder the conducive flow of ecosystem services, and the urban dwellers who are the beneficiaries will suffer from an interruption to services due to climatic and environmental changes [89, 90].

Increasing urban green spaces and green corridors along riverbanks and streets [91–94] is recognised more often in the actions list compared to other urban EbA measures. This finding could reflect a drive to expand greenery in the Kathmandu Valley since green spaces, including forests, parks, and green belts, are limited and comprise less than 4% of the total area of Kathmandu Metropolitan City [95]. Likewise, in a literature review of key studies on urban EbA, Brink et al. [3] have found that ecological structures (green space, wetlands, trees, and parks) were commonly leveraged to curb the risk of climate-related hazards in the urban sphere. Past scholarship has reported measures such as implementing green walls and roofs or facades to improve the thermal comfort of buildings [96–98] and unsealing and avoiding impervious surfaces to reduce stormwater runoff and prevent flooding and erosion [74]. Unfortunately, UHI was not explicitly mentioned in the policies and plans scrutinised in this study.

### 4.3 Appraisal of the integration of EbA measures across policies and plans

The breakdown of EbA mentions in the climate, urban, and sectoral policies and plans reveals varying levels of integration of EbA measures. The observed trends demonstrate the evolving recognition of EbA strategies within the examined policy and plan documents. The key finding for climate policies and plans is a discernible temporal pattern characterised by a gradual increase in the number of mentions of EbA. This result signifies a growing interest in EbA at the national level, which is very likely due to increasing awareness of its significance for climate change adaptation following its international endorsement and the influence of global agendas and commitments on climate action and nature-based interventions [45].

However, the integration of EbA is not satisfactory, especially for urban EbA measures. The NCCP [49] has the primary goal of ‘enhancing adaptive capacity and building ecosystem’s resilience at risk of adverse effects due to climate change’, but it fails to explicitly spell out measures to improve the resilience of urban ecosystems. This is a missed opportunity since the policy is the guiding document for the country’s climate action and could be anchored onto spearhead urban EbA initiatives. NAPA [50] is somewhat better in terms of referring to urban EbA measures and suggesting ecosystem-based interventions that are cross-sectoral and require multi-stakeholder engagement and transdisciplinary approaches, which are pre-requisites for implementing EbA measures [99].

Likewise, LAPA [51] aims to incorporate climate change adaptation and disaster risk reduction and management into local development priorities and natural resource management to improve ecosystem resilience, including in the urban domain. Efforts have also been invested in fostering a holistic approach to adaptation planning with the local institutions that are working to integrate LAPA with the Local Disaster Risk Management Planning, a local plan formulated with the support of the Ministry of Home Affairs [100]. This development is a stepping stone towards inter-ministerial coordination since Ministry of Forest and Environment primarily coordinates climate policy objectives at the federal, provincial, and local levels. However, there is a need to explore further ways to leverage ecosystem-based disaster risk reduction to accomplish integration and achieve the set targets. Ecosystem-based disaster risk reduction is a disaster risk reduction approach that harnesses the ecosystem and its services to prevent or mitigate the risk of natural hazards [101].

NAP [52] is the most progressive climate plan in urban EbA integration. This distinction can be attributed to the intersectoral working groups of nine thematic and cross-cutting groups guiding the NAP process, with the respective ministry responsible for a theme [102]. Out of the investigated urban policies and plans, SDMP [55] and NUDS [54] have the highest number of EbA-related measures in urban planning endeavours. These two documents were formulated around the same time—in 2015 and 2016, respectively—when climate change or global warming was already established as a pressing issue. NUDS adopts an intersectoral approach, with provisions to incorporate disaster risk management components into urban development plans and promote integrated safer settlement by prohibiting the development of urban infrastructures and settlements in risk-prone and environmentally sensitive areas [103]. Similarly, it can broaden its approach and incorporate Ecosystem-based disaster risk reduction components to seamlessly integrate ecosystem-based approaches into the existing policy framework and institutional mechanism. This path would be more pragmatic and efficient than crafting a completely different mechanism for EbA integration, which would only create policy confusion.

The urban plans demonstrate substantial incorporation of EbA measures. However, there is incoherency between urban policies and plans, as NUP [53] has limited content on ecosystem conservation and EbA and no evidence of measures for dealing with climate change issues. A possible reason for this is that NUP was established in 2007, when climate change and EbA had not yet been introduced into Nepal’s policy and planning processes. While NUP was a robust, forward-looking policy at the time of its formulation, it no longer reflects recent trends in sustainable urban development and climate change adaptation using nature-based solutions, particularly EbA in urban ecosystems. Thus, NUP requires a timely revision to tailor it to the current social, economic, and environmental challenges in urban areas. In addition, there is sectoral bias in the allocation of adaptation finances in Nepal, where urban settlements received only 0.01% of the funding between 2009 and 2014 [104]. Urban EbA should have a concrete positioning in NUP to change the status quo and provide an environment that enables it to flourish. Moreover, NUDS [54], SDMP [55], and RSLUP [56], which have relatively higher integration of urban EbA measures, still do not mention EbA in the *implementation* component. This aspect could be an entry point for the strategic integration of urban EbA to evade repercussions due to the absence of provisions and facilitate the generation of tangible outcomes for the urban environment and its residents.

Contrary to the urban plans, the sectoral policies and plans of NBSAP [59], FSS [61], WSSHSDP [62], DRRNSAP [58], and NEP [57] address EbA in a general context, and urban EbA-related content is either limited or non-existent. NBSAP recognises biodiversity services as cost-efficient and locally appropriate climate change adaptation measures, and it includes strategies for conserving landscapes, employing sustainable land practices, developing EbA programmes, and managing watershed degradation. Furthermore, it suggests a dedicated budget allocation to EbA within climate change funding, though it contains no specific mention of urban biodiversity. With the highest number of EbA mentions, FSS reflects an inherent connection to ecosystems, one of the two key components of EbA.

The forestry sector is heavily prioritised in Nepal, as evidenced by the allocation of about 45% of climate finances to the forestry and biodiversity sector for adaptation activities between 2009 and 2014 [104]. A report by the United Nations Development Programme [5] states that the forest sector covers 31% of EbA-related initiatives, the highest of all sectors, in Nepal. It can be extrapolated that Nepal employs integrated ecosystem-based approaches for forest adaptation plans to guide national development, thus underscoring the importance of ecosystem-focused strategies in mainstream development efforts [45]. However, it does not prioritise the issue of climate change and urban forests, which is consistent with the findings of Bhandari et al. [105]. While DRRNSAP does refer to guideline preparations for disaster risk reduction based on green infrastructure and EbA in the urban space, there is no clear indication of how ecosystem-based disaster risk reduction will be leveraged to support climate adaptation efforts in urban areas. Moreover, there is no explanation of which measures will be utilised or how resources will be procured and mobilised. NEP shows a similarly significant recognition of ecosystem protection and restoration and sustainable management of natural resources, with urban EbA limited to the development of parks and green belts alongside roads and rivers and the promotion of rainwater harvesting. WSSHSDP contains the fewest mentions of EbA, owing to its primary focus on basic service provision rather than direct integration of ecosystem-based approaches. Nevertheless, it does aim to maintain, protect, and regenerate ecosystems to increase water production in general. For a summary of the EbA-related content in each policy or plan along with the gaps in addressing urban EbA, see S1 Table.

The lack of urban EbA components in the sectoral plans and policies signals that they are not sensitive to the vital role of green and blue infrastructures and urban ecosystem services in sustaining and maintaining the ecological balance of the built environment and improving the quality of life of urban dwellers amidst the growing concerns of climate change [106]. The deficiency and disparity in integration of EbA measures also proves that sectoral policies often target a single sector without ensuring alignment with other sectors and climate issues. Evaluating policy coherence is crucial across levels—both vertically (from national to local levels) and horizontally (across line ministries and sectors). This approach enhances synergies and minimises conflict by identifying shared priorities, cross-cutting themes, and effective methods while enhancing monitoring and evaluation of progress, identifying potential partners and actors, and optimising local resources [107]. It is imperative to understand how EbA addresses climate issues in a particular ecosystem across multiple sectors. For effective integration and implementation of EbA, sectoral policies must introduce cross-sectoral actions and approaches that produce co-benefits for climate change adaptation and socio-economic development [108].

Despite the existing gaps in urban EbA, the inclusion of EbA measures across the majority of the analysed policies and plans evidences that Nepal has embraced EbA in policy and planning. Such inclusion reflects the unwavering commitment of Nepal to international agreements and initiatives, such as the Convention on Biological Diversity, the Paris Agreement, the 2030 Agenda for Sustainable Development Goals, the Sendai Framework for Disaster Risk Reduction, and the United Nations Decade on Ecosystem Restoration. These commitments underscore Nepal’s recognition of the pivotal role of the ecosystem in climate change adaptation and mitigation, disaster risk reduction, and sustainable development as well as the nation’s dedication to conserving ecosystems and biodiversity and enhancing resilience to climate change impacts for the benefit of nature and humans. The main challenge is to integrate these diverse efforts coherently to achieve sustainable impacts.

This study has its own set of limitations that could be addressed and improved upon in future research. The primary limitation lies in the selective inclusion of plans and policies, wherein all policies and plans could not be accommodated. Additionally, the urban plans and policies were restricted to the Kathmandu Valley. To delve deeper into urban EbA literature, future research could expand its scope by incorporating urban plans from different municipalities across Nepal, thereby observing trends and comparing differences in how urban EbA is addressed in the country. Further, the varying structures of plans and policies pose a challenge for comparative analysis. Future research may consider conducting individual studies on policies and plans separately, offering a more cohesive discussion and facilitating the development of a roadmap for EbA integration. The research applied deductive content analysis, deviating from strict adherence to explicit references to EbA, and considered any mention of ecosystems as indicative of EbA measures. Despite efforts to minimize personal bias, a degree of subjectivity may persist. Future researchers could enhance the qualitative scoring system by adopting more objective criteria. A balanced approach, integrating qualitative and quantitative measures, may enhance assessment robustness. Combining qualitative methods with quantitative measures, where applicable, can alleviate some of these drawbacks and contribute to a more comprehensive evaluation. Additionally, future research endeavors should include stakeholder mapping and analysis, crucial for prioritizing relevant and necessary EbA initiatives on the ground. This approach aids in tailoring engagement strategies, fostering local ownership, and amplifying the impact of EbA measures [109]. Furthermore, delving into stakeholders’ perceptions assumes a pivotal role in informing decision-making processes, revealing capacity gaps and needs among key actors [110]. This information not only informs policymakers but also sensitizes the policy development process to the merits of urban EbA, thereby contributing to its mainstreaming in Nepal.

## 5. Conclusion and policy recommendations

Nepal is making efforts to align its climate adaptation interventions with the global agendas and burgeoning interest in EbA. Through the in-depth examination of types of EbA measures and EbA-related information across plan components, this research reveals that Nepal’s policies and plans prioritise ecosystem conservation, restoration, and management to cope with the impacts of climate change to a certain extent. The EbA measures are predominantly incorporated into the *actions* component, which implies an action-oriented approach to mainstreaming EbA. However, the lack of a clearly defined pathway in the policies and plans for EbA implementation may cause ambiguity in operationalising the ambitious objectives and actionable strategies. The findings also uncover the urgent need to explicitly incorporate EbA measures to regulate the temperature and curb UHI, which are underexplored aims in the policies and plans sampled in this study. Another key finding is the disparity in integrating EbA across climate, urban, and sectoral policies and plans and the alarmingly scant focus on urban EbA measures to address climate change issues. While urban EbA integration appears to be emerging, it must be exhaustively incorporated to unlock the full potential of EbA and ecosystem-based disaster risk reduction in urban environments.

Despite this grim scenario, there is an opportunity to improve existing policies and plans and ensure the seamless integration of EbA through the strategic entry points identified in this study. Prompt actions must be taken to overcome the deficit of policy provisions and propel EbA measures in the urban sphere. Precursors to effective integration and implementation at the policy level include a proper understanding of the value of ecosystem-based approaches, the establishment of a clear *vision and objectives*, the definition of feasible actions tailored to the local context, and the integration of an inter-sectoral approach with clearly designated roles and responsibilities for the concerned government agency. These elements should be complemented by the capacity-building of different tiers of government institutions, the mobilisation of resources, the endorsement of multi-level governance and inter-ministerial coordination, and a robust institutional framework to support EbA mainstreaming. Climate interventions often face budget constraints from the government, which forces them to depend heavily on international donor agencies. This reliance hinders the integration of EbA into planning processes at national and sub-national levels. Therefore, the following policy recommendations are suggested to mainstream EbA into the policy landscape.

- Establish a comprehensive legislative framework explicitly integrating EbA concepts into urban planning, emphasizing the importance of nature-based solutions in climate resilience.
- Broaden the scope of EbA measures to include strategies addressing temperature regulation, mitigating the Urban Heat Island (UHI) effect, and enhancing thermal comfort in urban areas. Diversification of EbA measures unlock the co-benefits, notably the reduction in energy consumption, enhanced energy efficiency, and a consequential decrease in greenhouse gas emissions.
- Strengthen the operationalization of EbA by providing information on budget allocation, responsible bodies, project duration, etc. This enhancement requires an intricate understanding of ecosystem-based approaches, coupled with establishing a clear vision and defining locally-tailored feasible actions.
- Address institutional challenges arising from the federal system transition, fostering inter-ministerial coordination and building the capacity of provincial and local governments for effective EbA implementation in addition to clearly designating the roles and responsibilities of the concerned government agency.
- Revise outdated policies to align with current sustainable urban development and climate change adaptation trends, ensuring incorporation of urban EbA measures.
- Integrate urban EbA components into sectoral policies and encourage cross-sectoral actions and approaches within the sectoral policies, ensuring co-benefits for climate change adaptation and socio-economic development.
- Emphasize policy coherence across levels (national to local) and sectors, promoting synergies and minimizing conflicts by identifying shared priorities and cross-cutting themes to harmonize the approach towards EbA integration.

## Supporting information

S1 Table Summary of the EbA-related content in each policy/plan. The table contains breakdown of the EbA-related content both for general or urban reference (where relevant), along with the gaps in addressing urban EbA.
(DOCX)

S1 File Dataset. The file contains the data of the frequency distribution of EbA related content across the plan components and plans and policies, the breadth score for the EbA measures and the quality score of EbA integration.
(XLSX)

## References

[1] CBD Convention on Biological Diversity 2009: Connecting biodiversity and climate change mitigation and adaptation. Report of the second ad hoc technical expert group on biodiversity and climate change. CBD Technical Series No. 41. Secretariat of the Convention on Biological Diversity, Montreal, Canada; 2009.

[2] Munang R, Thiaw I, Alverson K, Mumba M, Liu J, Rivington M. Climate change and Ecosystem-based Adaptation: A new pragmatic approach to buffering climate change impacts. Current Opinion in Environmental Sustainability. 2013 Mar;5(1):67–71. doi: 10.1016/j.cosust.2012.12.001

[3] Brink E, Aalders T, Ádám D, Feller R, Henselek Y, Hoffmann A, et al. Cascades of green: A review of ecosystem-based adaptation in urban areas. Glob Environ Change. 2016 Jan 1;36:111–23. doi: 10.1016/j.gloenvcha.2015.11.003

[4] IUCN IUCN Global Standard for Nature-Based Solutions: A User-Friendly Framework for the Verification, Design and Scaling up of NbS (1st ed); 2020.

[5] UNDP Making the case for ecosystem-based adaptation: the global mountain EbA programme in Nepal, Peru and Uganda: The global mountain EBA Programme in Nepal, Peru and Uganda; 2015.

[6] Stergios-Aristoteles M, Dan VB, Sotirios A. Sustainability and climate resilience metrics and trade-offs in transport infrastructure asset recovery, Transportation Research Part D: Transport and Environment, Volume 121, 2023, 103800. doi: 10.1016/j.trd.2023.103800

[7] Kantartzis A, Malesios C, Stergiadou A, Theofanous N, Tampekis S, Arabatzis G. A Geographical Information Approach for Forest Maintenance Operations with Emphasis on the Drainage Infrastructure and Culverts. Water. 2021 May 18;13(10):1408. doi: 10.3390/w13101408

[8] IPCC Summary for policymakers [H.-O. Pörtner, D.C. Roberts, E.S. Poloczanska, K. Mintenbeck, M. Tignor, A. Alegría, M. Craig, S. Langsdorf, S. Löschke, V. Möller, A. Okem (eds.)]. In: Climate Change 2022: Impacts, adaptation and vulnerability. Contribution of working group ii to the sixth assessment report of the Intergovernmental Panel on Climate Change [H.-O. Pörtner, D.C. Roberts, M. Tignor, E.S. Poloczanska, K. Mintenbeck, A. Alegría, M. Craig, S. Langsdorf, S. Löschke, V. Möller, A. Okem, B. Rama (eds.)]. Cambridge, UK and New York: Cambridge University Press; 2022, p.3–33. 10.1017/9781009325844.001

[9] Pedersen Zari M. Regenerative urban design and ecosystem biomimicry. Oxon: Routledge; 2018.

[10] Kyriakopoulos GL, Sebos I. Enhancing Climate Neutrality and Resilience through Coordinated Climate Action: Review of the Synergies between Mitigation and Adaptation Actions. Climate. 2023 May 10;11(5):105. doi: 10.3390/cli11050105

[11] Doswald N, Osti M. Ecosystem-based approaches to adaptation and mitigation: Good practice examples and lessons learned in Europe. Deutschland/Bundesamt für Naturschutz; 2011.

[12] Roberts D, Boon R, Diederichs N, Douwes E, Govender N, Mcinnes A, et al. Exploring ecosystem-based adaptation in Durban, South Africa: “learning-by-doing” at the local government coal face. Environ Urban. 2012 Apr 1;24(1):167–95. doi: 10.1177/0956247811431412

[13] Jones HP, Hole DG, Zavaleta ES. Harnessing nature to help people adapt to climate change. Nat Clim Change. 2012 Jul;2(7):504–9. doi: 10.1038/nclimate1463

[14] Munang R, Thiaw I, Alverson K, Liu J, Han Z. The role of ecosystem services in climate change adaptation and disaster risk reduction. Curr Opin Environ Sustain. 2013 Mar 1;5(1):47–52. doi: 10.1016/j.cosust.2013.02.002

[15] Munang R, Thiaw I, Alverson K, Goumandakoye M, Mebratu D, Liu J. Using ecosystem-based adaptation actions to tackle food insecurity. Environment: Science and Policy for Sustainable Development. 2013c. Jan; 55(1): 29–35.

[16] Doswald N, Munroe R, Roe D, Giuliani A, Castelli I, Stephens J, et al. Effectiveness of ecosystem-based approaches for adaptation: review of the evidence-base. Clim Dev. 2014 Apr 3;6(2):185–201. doi: 10.1080/17565529.2013.867247

[17] McVittie A, Cole L, Wreford A, Sgobbi A, Yordi B. Ecosystem-based solutions for disaster risk reduction: Lessons from European applications of ecosystem-based adaptation measures. Int J Disaster Risk Reduct. 2018 Dec 1;32:42–54. doi: 10.1016/j.ijdrr.2017.12.014

[18] Zölch T, Wamsler C, Pauleit S. Integrating the ecosystem-based approach into municipal climate adaptation strategies: The case of Germany. J Clean Prod. 2018;170:966–77. doi: 10.1016/j.jclepro.2017.09.146

[19] Sandholz S, Lange W, Nehren U. Governing green change: Ecosystem-based measures for reducing landslide risk in Rio de Janeiro. Int J Disaster Risk Reduct. 2018 Dec 1;32:75–86. doi: 10.1016/j.ijdrr.2018.01.020

[20] Pedersen Zari M, Blaschke PM, Jackson B, Komugabe-Dixson A, Livesey C, Loubser DI, et al. Devising urban ecosystem-based adaptation (EbA) projects with developing nations: A case study of Port Vila, Vanuatu. Ocean Coast Manag. 2020 Feb 1;184:105037. doi: 10.1016/j.ocecoaman.2019.105037

[21] Goodwin S, Olazabal M, Castro AJ, Pascual U. Global mapping of urban nature-based solutions for climate change adaptation. Nat Sustain. 2023 Apr;6(4):458–69. doi: 10.1038/s41893-022-01036-x

[22] Vignola R, Locatelli B, Martinez C, Imbach P. Ecosystem-based adaptation to climate change: what role for policy-makers, society and scientists? Mitig Adapt Strateg Glob Change. 2009 Dec 1;14(8):691–6. doi: 10.1007/s11027-009-9193-6

[23] Huq N, Renaud F, Sebesvari Z. Ecosystem Based Adaptation (EbA) to climate change-integrating actions to sustainable adaptation. United Nations University-Institute for Environment and Human Security (UNU-EHS). 2013.

[24] Scarano FR. Ecosystem-based adaptation to climate change: concept, scalability and a role for conservation science. Perspectives in Ecology and Conservation. 2017 Apr;15(2):65–73. doi: 10.1016/j.pecon.2017.05.003

[25] Uittenbroek CJ, Janssen-Jansen LB, Runhaar HAC. Mainstreaming climate adaptation into urban planning: overcoming barriers, seizing opportunities and evaluating the results in two Dutch case studies. Reg Environ Change. 2013 Apr 1;13(2):399–411. doi: 10.1007/s10113-012-0348-8

[26] Wamsler C, Luederitz C, Brink E. Local levers for change: Mainstreaming ecosystem-based adaptation into municipal planning to foster sustainability transitions. Global Environmental Change. 2014 Nov;29:189–201. doi: 10.1016/j.gloenvcha.2014.09.008

[27] Wamsler C, Pauleit S. Making headway in climate policy mainstreaming and ecosystem-based adaptation: two pioneering countries, different pathways, one goal. Clim Change. 2016 Jul 1;137(1):71–87. doi: 10.1007/s10584-016-1660-y

[28] Schneider P, Walz A, Albert C, Lipp T. Ecosystem-based adaptation in cities: Use of formal and informal planning instruments. Land Use Policy. 2021 Oct 1;109:105722. doi: 10.1016/j.landusepol.2021.105722

[29] IUCN The Ecosystem Approach: Learning from Experience; Shepherd, G., Ed. Gland, Switzerland: IUCN; 2008.

[30] Colls A, Ash N, Ikkala N. Ecosystem-based adaptation: A natural response to climate change. Gland, Switzerland: IUCN; 2009.

[31] Huq N, Bruns A, Ribbe L, Huq S. Mainstreaming Ecosystem Services Based Climate Change Adaptation (EbA) in Bangladesh: Status, Challenges and Opportunities. Sustainability. 2017 Jun;9(6):926. doi: 10.3390/su9060926

[32] Eckstein D, Hutfils M, Winges M. Global Climate Risk Index. Germanwatch, Bonn, Germany; 2019. Available at https://germanwatch.org/en/cri.

[33] UNDESA World Urbanization Prospects: 2014 Revision. New York: United Nations Department of Economic and Social Affairs; 2014. Available from: http://esa.un.org/unpd/wup/FinalReport/WUP2014-Report.pdf

[34] World Bank. Population growth (annual%) Nepal [online]; 2021. Available from: https://data.worldbank.org/indicator/SP.POP.GROW?locations=NP

[35] National Statistics Office. National Population and Housing Census 2021: National Report. Government of Nepal, Office of the Prime Minister and Council of Ministers, National Statistics Office, Thapathali, Kathmandu, Nepal; 2021.

[36] Ishtiaque A, Shrestha M, Chhetri N. Rapid urban growth in the Kathmandu Valley, Nepal: Monitoring land use land cover dynamics of a Himalayan city with landsat imageries. Environments. 2017 Oct 8;4(4):72. Available from: https://www.mdpi.com/2076-3298/4/4/72/pdf

[37] KC S, Shrestha S, Ninsawat S, Chonwattana S. Predicting flood events in Kathmandu Metropolitan City under climate change and urbanisation. Journal of Environmental Management. 2021 Mar;281:111894. doi: 10.1016/j.jenvman.2020.111894 33412359

[38] Mishra B, Sandifer J, Gyawali BR. Urban Heat Island in Kathmandu, Nepal: Evaluating Relationship between NDVI and LST from 2000 to 2018. International Journal of Environment. 2019 Jan 30;8(1):17–29. doi: 10.3126/ije.v8i1.22546

[39] Ministry of Population and Environment (MOPE). Vulnerability and Risk Assessment Framework and Indicators for National Adaptation Plan (NAP) Formulation Process in Nepal. Kathmandu: Government of Nepal; 2017. Available from: https://floodresilience.net/resources/item/vulnerability-and-risk-assessment-framework-andindicators-for-national-adaptation-plan-nap-formulation-process-in-nepal.

[40] Adhikari S, Baral H, Nitschke C. Adaptation to Climate Change in Panchase Mountain Ecological Regions of Nepal. Environments. 2018 Mar;5(3):42. doi: 10.3390/environments5030042

[41] Reid H, Adhikari A. Ecosystem-based approaches to adaptation: strengthening the evidence and informing policy. Research results from the Mountain EbA Project, Nepal. Ecosyst-Based Approaches Adapt Strength Evid Informing Policy Res Results Mt EbA Proj Nepal. 2018 [cited 2023 Jun 28]; Available from: https://www.cabdirect.org/cabdirect/abstract/20183269662.

[42] Fu C, Vijitpan T, Bogati R, Shrestha TK, Wang G. Participatory Process for Implementing Ecosystem-based Adaptation in a Mountainous District of Nepal. In: Leal Filho W, Luetz J, Ayal D, editors. Handbook of Climate Change Management: Research, Leadership, Transformation. Cham: Springer International Publishing; 2020 [cited 2023 Jun 28]. p. 1–16. 10.1007/978-3-030-22759-3_331-1

[43] Karki G, Bhatta B, Devkota NR, Acharya RP, Kunwar RM. Climate Change Adaptation (CCA) Interventions and Indicators in Nepal: Implications for Sustainable Adaptation. Sustainability. 2021 Jan;13(23):13195. doi: 10.3390/su132313195

[44] Bhattarai S, Regmi BR, Pant B, Uprety DR, Maraseni T. Sustaining ecosystem based adaptation: The lessons from policy and practices in Nepal. Land Use Policy. 2021 May 1;104:105391. doi: 10.1016/j.landusepol.2021.105391

[45] Poudel S, Mishra B, Shaw R. Ecosystem-Based Approaches and Policy Perspectives in Nepal. Disaster and risk research: GADRI book series. 2021 Jan 1;85–100.

[46] Ahern J, Cilliers S, Niemelä J. The concept of ecosystem services in adaptive urban planning and design: A framework for supporting innovation. Landsc Urban Plan. 2014 May 1;125:254–9. doi: 10.1016/j.landurbplan.2014.01.020

[47] Sandholz S. Potential for Ecosystem-Based Disaster Risk Reduction and Climate Change Adaptation in the Urban Landscape of Kathmandu Valley, Nepal. In: Renaud FG, Sudmeier-Rieux K, Estrella M, Nehren U, editors. Ecosystem-Based Disaster Risk Reduction and Adaptation in Practice. Cham: Springer International Publishing; 2016. p. 335–60. (Advances in Natural and Technological Hazards Research). 10.1007/978-3-319-43633-3_15

[48] Cortinovis C, Geneletti D. Ecosystem services in urban plans: What is there, and what is still needed for better decisions. Land Use Policy. 2018 Jan 1;70:298–312. doi: 10.1016/j.landusepol.2017.10.017

[49] National Climate Change Policy (NCCP). Ministry of Forest and Environment, Government of Nepal; 2019. Available from: https://climate.mohp.gov.np/31-acts/153-climate-change-policy.

[50] National Adaptation Programme of Action to Climate Change (NAPA). Ministry of Environment, Government of Nepal; 2010. Available from: https://www.climatenepal.org.np/project/national-adaptation-programme-action-napa.

[51] Local Adaptation Plans for Action (LAPA). Ministry of Forests and Environment, Government of Nepal; 2019.

[52] National Adaptation Plan (NAP). Ministry of Forests and Environment, Government of Nepal; 2021. Available from: https://www.mofe.gov.np/uploads/uploads/notices/nap-full-repnoticepdf-3463-7921661679518.pdf.

[53] National Urban Policy (NUP). Department of Urban Development and Building Construction. Ministry of Physical Planning and Works, Government of Nepal; 2007. Available from: https://www.moud.gov.np/storage/listies/May2023/national-urban-policy-2007-compressed.pdf.

[54] National Urban Development Strategy (NUDS). Urban Development and Physical Planning Division. Ministry of Urban Development, Government of Nepal; 2017. Available from: https://www.moud.gov.np/storage/listies/July2019/NUDS_PART_A.pdf.

[55] KVDA. 20 years Strategic Development Master Plan (SDMP) for Kathmandu Valley. Kathmandu Valley Development Authority (KVDA), Ministry of Urban Development, Government of Nepal; 2015. Available from: http://www.kvda.gov.np/uploads/form/SDMP%20part1.pdf.

[56] Risk Sensitive Land Use Plan of Kathmandu Valley (RSLUP). Kathmandu Valley Development Authority, Ministry of Urban Development, Government of Nepal; 2016.

[57] National Environment Policy (NEP). Ministry of Forests and Environment, Government of Nepal; 2019. Available from: https://www.mofe.gov.np/uploads/documents/national-environment-policy1563366482pdf-2660-693-1658746861.pdf

[58] Disaster Risk Reduction National Strategic Action Plan (DRRNSAP). Ministry of Home Affairs, Government of Nepal; 2018. Available from: http://drrportal.gov.np/document/documentdetail/1352

[59] National Biodiversity Strategy and Action Plan (NBSAP). Ministry of Forests and Soil Conversation, Government of Nepal; 2014. Available from: https://www.cbd.int/doc/world/np/np-nbsap-v2-en.pdf.

[60] National Land Use Policy (NLUP). Ministry of Land Reform and Management, Government of Nepal; 2015. https://molcpa.gov.np/downloadfile/land%20use%20policy_2015_1505895657_1536124080.pdf

[61] Forest Sector Strategy (FSS). Ministry of Forests and Soil Conversation, Government of Nepal; 2016. Available from: https://www.mofe.gov.np/uploads/documents/forestry-sector-strategy-2016-20251526466721pdf-7869-771-1658747252.pdf.

[62] Water Supply, Sanitation and Hygiene Sector Development Plan (WSSHSDP). Ministry of Water Supply and Sanitation, Government of Nepal; 2016. Available from: https://www.fsmtoolbox.com/assets/pdf/25._SDP_-_Final-_Eng.pdf.

[63] Gómez-Baggethun E, Barton DN. Classifying and valuing ecosystem services for urban planning. Ecol Econ. 2013 Feb 1;86:235–45. doi: 10.1016/j.ecolecon.2012.08.019

[64] Geneletti D, Zardo L. Ecosystem-based adaptation in cities: An analysis of European urban climate adaptation plans. Land Use Policy. 2016 Jan 1;50:38–47. doi: 10.1016/j.landusepol.2015.09.003

[65] EEA. Urban adaptation to climate change in Europe Challenges and opportunities for cities together with supportive national and European policies. European Environmental Agency. EEA Technical report No 2/2012; 2012; p.143.

[66] Bolund P, Hunhammar S. Ecosystem services in urban areas. Ecol Econ. 1999;29(2):293–301. doi: 10.1016/S0921-8009(99)00013-0

[67] Benedict M, McMahon E, Fund T, Bergen L. Green Infrastructure: Linking Landscapes and Communities. Bibliovault OAI Repos Univ Chic Press. 2006 Jan;22

[68] Heidrich O, Dawson RJ, Reckien D, Walsh CL. Assessment of the climate preparedness of 30 urban areas in the UK. Climatic Change. 2013 Jul 27;120(4):771–84. doi: 10.1007/s10584-013-0846-9

[69] Baker I, Peterson A, Brown G, McAlpine C. Local government response to the impacts of climate change: An evaluation of local climate adaptation plans. Landsc Urban Plan. 2012 Aug 1;107(2):127–36. doi: 10.1016/j.landurbplan.2012.05.009

[70] Rozas-Vásquez D, Fürst C, Geneletti D, Almendra O. Integration of ecosystem services in strategic environmental assessment across spatial planning scales. Land Use Policy. 2018 Feb;71:303–10. doi: 10.1016/j.landusepol.2017.12.015

[71] Bruno E, Falco E, Shahab S, Geneletti D. Integrating ecosystem services in transfer of development rights: a literature review. Land Use Policy. 2023 Aug 1;131:106694. doi: 10.1016/j.landusepol.2023.106694

[72] Hsieh HF, Shannon SE. Three Approaches to Qualitative Content Analysis. Qual Health Res. 2005 Nov 1;15(9):1277–88. doi: 10.1177/1049732305276687 16204405

[73] Braat LC, de Groot R. The ecosystem services agenda:bridging the worlds of natural science and economics, conservation and development, and public and private policy. Ecosyst Serv. 2012 Jul 1;1(1):4–15. doi: 10.1016/j.ecoser.2012.07.011

[74] Tang Z, Brody SD, Quinn C, Chang L, Wei T. Moving from agenda to action: evaluating local climate change action plans. J Environ Plan Manag. 2010 Jan 1;53(1):41–62. doi: 10.1080/09640560903399772

[75] Kumar P, Geneletti D. How are climate change concerns addressed by spatial plans? An evaluation framework, and an application to Indian cities. Land Use Policy. 2015 Jan 1;42:210–26. doi: 10.1016/j.landusepol.2014.07.016

[76] Koo TK, Li MY. A Guideline of Selecting and Reporting Intraclass Correlation Coefficients for Reliability Research. J Chiropr Med. 2016 Jun;15(2):155–63. doi: 10.1016/j.jcm.2016.02.012 27330520 PMC4913118

[77] Bouwma I, Schleyer C, Primmer E, Winkler KJ, Berry P, Young J, et al. Adoption of the ecosystem services concept in EU policies. Ecosyst Serv. 2018;29:213–22. doi: 10.1016/j.ecoser.2017.02.014

[78] Wamsler C. Mainstreaming ecosystem-based adaptation: transformation toward sustainability in urban governance and planning Ecol Soc. 2015;20(2). Available from: https://www.jstor.org/stable/26270196. doi: 10.5751/ES-07489-200230

[79] Pauleit S, Zölch T, Hansen R, Randrup TB, Konijnendijk van den Bosch C. Nature-Based Solutions and Climate Change—Four Shades of Green. Theory and Practice of Urban Sustainability Transitions. 2017;29–49. doi: 10.1007/978-3-319-56091-5_3

[80] Oke TR. Street design and urban canopy layer climate. Energy Build. 1988;11(1):103–13. doi: 10.1016/0378-7788(88)90026-6

[81] Hsieh CM, Huang HC. Mitigating urban heat islands: A method to identify potential wind corridor for cooling and ventilation. Comput Environ Urban Syst. 2016;57:130–43. doi: 10.1016/j.compenvurbsys.2016.02.005

[82] Ribeiro RM, Amaral S, Monteiro AMV, Dal’Asta AP. “Cities in the forest” and “cities of the forest”: An environmental Kuznets curve (EKC) spatial approach to analyzing the urbanization-deforestation relationship in a Brazilian Amazon state. Ecology and Society. 2022;27(2). doi: 10.5751/ES-13224-270201

[83] Sebos I, Kitsara G, Karali A, Giannakopoulos A, Chioti D, Katsaros A, et al. Climate-resilient urban regeneration: Transforming school yards for a sustainable and adaptive future. EMS Annual Meeting 2023, Bratislava, Slovakia, 4–8 Sep 2023, EMS2023-73. 10.5194/ems2023-73

[84] Sofios S, Arabatzis G, Baltas E. Policy for management of water resources in Greece. The Environmentalist. 2007 Aug 14;28(3):185–94. *** change reference number doi: 10.1007/s10669-007-9126-4

[85] Acharya KK. Local Governance Restructuring in Nepal: From Government to Governmentality. Dhaulagiri J Sociol Anthropol. 2018 Dec 31;12:37–49. doi: 10.3126/dsaj.v12i0.22178

[86] Darjee KB, Sunam RK, Köhl M, Neupane PR. Do National Policies Translate into Local Actions? Analyzing Coherence between Climate Change Adaptation Policies and Implications for Local Adaptation in Nepal. Sustainability. 2021 Jan;13(23):13115. doi: 10.3390/su132313115

[87] Khatri DB, Nightingale AJ, Ojha H, Maskey G, Lama ‘Tsumpa’ PN. Multi-scale politics in climate change: the mismatch of authority and capability in federalizing Nepal. Clim Policy. 2022 Sep 14;22(8):1084–96. doi: 10.1080/14693062.2022.2090891

[88] Geneletti D, Cortinovis C, Zardo L, Adem Esmail B. Reviewing Ecosystem Services in Urban Climate Adaptation Plans. In: Planning for Ecosystem Services in Cities. Cham: Springer International Publishing; 2020. p. 21–30. 10.1007/978-3-030-20024-4_3

[89] Dearborn DC, Kark S. Motivations for Conserving Urban Biodiversity. Conserv Biol. 2010;24(2):432–40. doi: 10.1111/j.1523-1739.2009.01328.x 19775276

[90] Taylor L, Hochuli DF. Creating better cities: how biodiversity and ecosystem functioning enhance urban residents’ wellbeing. Urban Ecosyst. 2015 Sep 1;18(3):747–62. doi: 10.1007/s11252-014-0427-3

[91] Bowler DE, Buyung-Ali L, Knight TM, Pullin AS. Urban greening to cool towns and cities: A systematic review of the empirical evidence. Landscape and Urban Planning. 2010 Sep;97(3):147–55. doi: 10.1016/j.landurbplan.2010.05.006

[92] Niemelä J, Saarela SR, Söderman T, Kopperoinen L, Yli-Pelkonen V, Väre S, et al. Using the ecosystem services approach for better planning and conservation of urban green spaces: a Finland case study. Biodiversity and Conservation. 2010 Jul 7;19(11):3225–43. Available from: https://link.springer.com/article/10.1007%2Fs10531-010-9888-8.

[93] Govindarajulu D. Urban green space planning for climate adaptation in Indian cities. Urban Clim. 2014 Dec 1;10:35–41. doi: 10.1016/j.uclim.2014.09.006

[94] Schmidt K, Walz A. Ecosystem-based adaptation to climate change through residential urban green structures: co-benefits to thermal comfort, biodiversity, carbon storage and social interaction. One Ecosyst. 2021 Dec 16;6:e65706. doi: 10.3897/oneeco.6.e65706

[95] Pokharel S. Green space suitability evaluation for urban resilience: an analysis of Kathmandu Metropolitan city, Nepal. Environmental Research Communications. 2019 Oct 8;1(10):105003. doi: 10.1088/2515-7620/ab4565

[96] Alexandri E, Jones P. Temperature decreases in an urban canyon due to green walls and green roofs in diverse climates. Build Environ. 2008 Apr 1;43(4):480–93. doi: 10.1016/j.buildenv.2006.10.055

[97] Wong MS, Nichol JE, To PH, Wang J. A simple method for designation of urban ventilation corridors and its application to urban heat island analysis. Build Environ. 2010 Aug 1;45(8):1880–9. doi: 10.1016/j.buildenv.2010.02.019

[98] Skelhorn C, Lindley S, Levermore G. The impact of vegetation types on air and surface temperatures in a temperate city: A fine scale assessment in Manchester, UK. Landsc Urban Plan. 2014 Jan 1;121:129–40. doi: 10.1016/j.landurbplan.2013.09.012

[99] Wertz-Kanounnikoff S, Locatelli B, Wunder S, Brockhaus M. Ecosystem-based adaptation to climate change: What scope for payments for environmental services? Clim Dev. 2011 Apr 1;3(2):143–58. doi: 10.1080/17565529.2011.582277

[100] Ghimire R, Chhetri N. Challenges and prospects of Local Adaptation Plans of Action (LAPA) initiative in Nepal as everyday adaptation. Ecol Soc. 2022 Dec 26;27(4). Available from: https://ecologyandsociety.org/vol27/iss4/art28/.

[101] Dissanayaka KDCR, Tanaka N, Vinodh TLC. Integration of Eco-DRR and hybrid defense system on mitigation of natural disasters (Tsunami and Coastal Flooding): a review. Nat Hazards. 2022 Jan 1;110(1):1–28. doi: 10.1007/s11069-021-04965-6

[102] MoFE. Nepal’s National Adaptation Plan (NAP) Process: Reflecting on Lessons Learned and the Way Forward; 2018. Available from: http://napglobalnetwork.org/wp-content/uploads/2018/07/napgn-en-2018-nepal-nap-process.pdf.

[103] Timsina NP, Shrestha A, Poudel DP, Upadhyaya R. Trend of urban growth in Nepal with a focus in Kathmandu Valley: A review of processes and drivers of change. 2020 Sep. Available from: https://era.ed.ac.uk/handle/1842/37436.

[104] Oxfam. Finding the money: A stock taking of climate change adaptation finance and governance in Nepal; 2014. Oxfam, Country Office, Nepal, Lalitpur.

[105] Bhandari A, Thakuri S, Koirala P, Devkota M. Conflict-Sensitive Climate Change Adaptation in Nepal: An Analysis of Climate Resilience Policy Frameworks. Journal of Forest and Livelihood. 2021;20(1): 31–44. Available from: https://forestaction.org/wp-content/uploads/2022/03/Bhandari-et-al.pdf.

[106] Tzoulas K, Korpela K, Venn S, Yli-Pelkonen V, Kaźmierczak A, Niemela J, et al. Promoting ecosystem and human health in urban areas using Green Infrastructure: A literature review. Landsc Urban Plan. 2007 Jun 20;81(3):167–78. doi: 10.1016/j.landurbplan.2007.02.001

[107] Joshi GR, Joshi B. Agricultural and Natural Resources Policies in Nepal: A Review of Formulation and Implementation Processes and Issues. Nepal Public Policy Rev. 2021 Sep 18;1:212–27. doi: 10.3126/nppr.v1i1.43459

[108] Nguyen TT, Pittock J, Huong N. Integration of ecosystem-based adaptation to climate change policies in Viet Nam. Clim Change. 2017 Jan 1;97–111. doi: 10.1007/s10584-017-1936-x

[109] Ioanna N, Pipina K, Despina C, Ioannis S, Dionysis A. Stakeholder mapping and analysis for climate change adaptation in Greece. Euro-Mediterranean Journal for Environmental Integration. 2022 Sep;7(3):339–46. doi: 10.1007/s41207-022-00317-3

[110] Zerva A, Tsantopoulos G, Grigoroudis E, Arabatzis G. Perceived citizens’ satisfaction with climate change stakeholders using a multicriteria decision analysis approach. Environmental Science and Policy, 2018 Feb; 82:60–70. doi: 10.1016/j.envsci.2018.01.008

