# Supplementary material for: Integration of urban ecosystem-based adaptation in Nepal: A policy landscape analysis
Source: PLoS One. 2024 Jan 31;19(1):e0297786. doi: 10.1371/journal.pone.0297786 (PMC10829995; doi:10.1371/journal.pone.0297786)
Supplement: S1 Table — The table contains breakdown of the EbA-related content both for general or urban reference (where relevant), along with the gaps in addressing urban EbA. (DOCX) [file pone.0297786.s001.docx]

S1 Table: A summary of EbA-related content for each policy and plan together with the gaps in addressing urban EbA

| S.N. | Policies/Plans | EbA-related content | | Addressing the urban EbA gaps |
| --- | --- | --- | --- | --- |
|  |  | General | Urban |  |
| 1. | National Climate Change Policy (NCCP) 2019 | - Includes developing and implementing nature-based technologies, piloting ecosystem-based adaptation programs, and exploring payment for ecosystem services | N/A | - Expand urban EbA measures by broadening the target area and accelerating uptake. - Establish a policy framework, institutional mechanisms, and allocate sufficient budget to create an enabling environment for mainstreaming EbA measures. |
| 2. | National Adaptation Programme of Action (NAPA) 2010, | - Recognizes the need for an integrated ecosystem approach to climate adaptation. - Implicit and explicit EbA actions are outlined in three key areas: water resources, energy, forest, biodiversity, and public health. - Proposes substantial EbA measures for rural and urban ecosystems | - Interventions related to urban EbA are heavily focused on watershed conservation and rainwater harvesting | - Expand the EbA measures to green infrastructures and formulate a concrete plan for the implementation of the specified strategies |
| 3. | Local Adaptation Plans for Action (LAPA) 2019 | - Integrates climate adaptation and disaster risk reduction into local development priorities. - Focuses on enhancing ecosystem resilience and sustainable resource management. - Promotes EbA programs in existing local plans | - Suggests adopting climate adaptation technologies in the design of urban infrastructures, applying green settlement concepts and tailoring local land use plans to cope with climate risks | - Need to provide details into how EbA can be leveraged to facilitate the integration of climate change adaptation and disaster risk reduction and management (Eco-DRR exploration is required) |
| 4. | National Adaptation Plan (NAP) 2021-2050 | - Prioritizes ecosystem integrity as a core principle, promoting nature-based solutions. - Emphasizes adaptation programs for forests, biodiversity, and watershed conservation to secure and enhance ecosystem services. | - Recommends integrated urban models for climate resilience through nature-based solutions but lacks concrete actions in project implementation. | - Elucidate the nature-based solutions that will be applied to deal with climate-related hazards and how they will be made feasible and operationalized |
| 5. | National Urban Policy (NUP) 2007 | - Endorses protecting and sustainably utilizing natural resources comprising of rivers, streams, watershed areas and forest areas and rainwater harvesting, without addressing climate change impacts | | - Revision needed to recognize urban ecosystem services, enhance tailored EbA measures for urban climate hazards, align with urban plans, and amplify overall policy and planning effectiveness. |
| 6. | National Urban Development Strategy (NUDS) 2016 | - Prioritizes greening cities for sustainable urban development. - Includes numerous EbA measures for green and blue infrastructure, such as open spaces, water protection, rainwater harvesting, eco-friendly construction, and urban forest promotion. However, lacks specific implementation measures. | | - Craft a concise blueprint for implementing set goals and strategies to achieve tangible outputs and outcomes. |
| 7. | Vision 2035 and Beyond: 20 years Strategic Development Master Plan (SDMP) 2015-2035 for Kathmandu Valley | - Encompasses diverse urban EbA measures but the implementation lacks clarity, with the operational plan pending development. | | - Prioritize expediting the operational plan formulation |
| 8. | Risk Sensitive Land Use Plan of Kathmandu Valley (RSLUP) 2016 | - Acknowledges the expansion of Risk-Sensitive Land Use Planning to include Eco-DRR. - Highlights EbA measures for flood control and stormwater runoff but lacks provisions for reducing the urban heat island effect and an implementation plan | | - Introduce temperature regulation measures and elaborate on the practical enforcement approach for the specified actions. |
| 9. | National Land Use Policy (NLUP) 2015 | - Incorporates greenery, green belts and open spaces into urban settings and aligns with land use plans and Building Code. - Supports sustainable urbanization and ecosystem conservation, optimizing land and resource use. | | - Create an intersectoral mechanism to foster cooperation among different line ministries, preventing silos and duplication of efforts |
| 10. | Disaster Risk Reduction National Strategic Action Plan (DRRNSAP) 2018-2030 | - Endorses alignment between disaster risk reduction, climate adaptation, and environmental management. - Calls for an Eco-DRR and Management Fund at all levels but lacks specifics on local-level channelization. | - Involves creating guidelines for urban disaster risk reduction using green infrastructure and EbA. | - Specify how Eco-DRR will contribute to urban climate adaptation efforts. - Clarify measures, resource procurement, and mobilization plans for implementation. |
| 11. | National Biodiversity Strategy and Action Plan (NBSAP) 2014-2020 | - Recognizes biodiversity services as cost-effective climate adaptation measures. Strategies include conserving landscapes, employing suitable land practices, and developing EbA programs for biodiversity conservation. - Proposes allocating a budget for EbA under climate change funding. | N/A | - Formulate strategies to conserve urban biodiversity for climate change. - Develop funding mechanisms for EbA projects. |
| 12. | National Environment Policy (NEP) 2019 | - Promotes ecosystem protection, sustainable resource management, rainwater harvesting, strategic tree planting along roads and rivers, and ecosystem restoration. | - Mention of urban EbA is limited to developing parks and increasing greenery | - Include urban environment, enhance green and blue infrastructure and emphasize their role in addressing climate change. |
| 13. | Forest Sector Strategy (FSS) 2016-2025 | - Recognizes forests and watersheds for climate adaptation, emphasizing interventions like landscape approaches and sustainable management, with aim to mainstream ecosystem-based approaches by 2025. | - Promotes urban forestry integration into planning through legal provisions, fostering open spaces, tree planting for amenities, and recreational facilities for a safer, greener urban environment. | - Ensure effective strategy implementation by providing well-defined details for operationalizing priority programs and themes. |
| 14. | Water Supply, Sanitation and Hygiene Sector Development Plan (WSSHSDP) 2016–2030 | - Aims to enhance water production by maintaining, protecting, and regenerating ecosystems. Involves measures like rainwater recharge, source protection, and a river health-monitoring program in the urban context. | | - Establish inter-sectoral and interagency coordination mechanisms to amplify the benefits of strategic actions shared with other sectoral plans (forestry, biodiversity, etc.). |
